# Plastid retrograde regulation of miRNA expression in response to light stress

**DOI:** 10.1186/s12870-022-03525-9

**Published:** 2022-03-26

**Authors:** Anna Barczak-Brzyżek, Grzegorz Brzyżek, Marek Koter, Ewa Siedlecka, Piotr Gawroński, Marcin Filipecki

**Affiliations:** 1 Department of Plant Genetics, Breeding and Biotechnology, Institute of Biology, Warsaw University of Life Sciences-SGGW, 02-776 Warsaw, Poland; 2 Institute of Biochemistry and Biophysics Polish Academy of Sciences, 02-106 Warsaw, Poland

**Keywords:** High light, miRNAs, Chloroplast, Singlet oxygen, Plastoquinone

## Abstract

**Background:**

MicroRNAs (miRNAs) are a class of endogenous noncoding RNAs that play a pivotal role in the regulation of plant development and responses to the surrounding environment. Despite the efforts made to elucidate their function in the adaptation of plants to many abiotic and biotic stresses, their role in high light (HL) stress is still vague. HL stress often arises upon plant exposure to full sunlight. Subsequent changes in nuclear gene expression are triggered by chloroplast-derived retrograde signals.

**Results:**

In this study, we show that HL is involved in miRNA-dependent regulation in *Arabidopsis thaliana* rosettes. Microtranscriptomic screening revealed a limited number of miRNAs reacting to HL. To explain the miRNA regulation mechanisms at the different biogenesis stages, chemical and genetic approaches were applied. First, we tested the possible role of plastoquinone (PQ) redox changes using photosynthetic electron transport chain inhibitors. The results suggest that increased primary transcript abundance (pri-miRNAs) of HL-regulated miRNAs is dependent on signals upstream of PQ. This indicates that such signals may originate from photosystem II, which is the main singlet oxygen (^1^O_2_) source. Nevertheless, no changes in pri-miRNA expression upon a dark–light shift in the conditional *fluorescent* (*flu*) mutant producing ^1^O_2_ were observed when compared to wild-type plants. Thus, we explored the ^1^O_2_ signaling pathway, which is initiated independently in HL and is related to β-carotene oxidation and production of volatile derivatives, such as β-cyclocitral (β-CC). Pri-miRNA induction by β-CC, which is a component of this ^1^O_2_ pathway, as well as an altered response in the *methylene blue sensitivity 1* (*mbs1*) mutant support the role of ^1^O_2_ signaling in miRNA regulation.

**Conclusions:**

We show that light stress triggers changes in miRNA expression. This stress response may be regulated by reactive oxygen species (ROS)-related signaling. In conclusion, our results link ROS action to miRNA biogenesis, suggesting its contribution to inconsistent pri- and mature miRNA dynamics.

**Supplementary Information:**

The online version contains supplementary material available at 10.1186/s12870-022-03525-9.

## Background

Plants take fundamental advantage of light absorption, but constant light fluctuations often result in episodes of excess light energy [1]. Many physiological and molecular processes are engaged in adjusting the plant response to light stress because of its frequent occurrence and possible consequences in photoinhibition [2]. Light-originated stimuli are perceived by chloroplasts and trigger retrograde signaling, resulting in nuclear gene expression changes [3–5]. MicroRNAs (miRNAs) are nucleus-encoded molecules that play an effective fine-tuning role in the plant response to environmental stresses [6]. Thus, the study of their contribution to the light stress response involving chloroplast-derived signals is extremely important. Moreover, miRNAs largely target transcription factors (TFs), which have paramount importance for plant growth, reproduction, and defense [7–9]. Surprisingly, knowledge concerning light-regulated miRNAs is limited. For example, far-red light-responsive miRNAs were described in soybean [10]. Nevertheless, there is almost no information about miRNA expression changes under high light (HL) conditions except studies on *Dendrocalamus latiflorus*, an important Asian bamboo species [11], a recent report describing small RNAs during high light acclimation [12] and the systemic miRNA response in *Arabidopsis thaliana* roots [13]. Additionally, there are some reports on UV-A-, UV-B-, and gamma radiation-regulated miRNAs [14–18].

Almost all *MIR* genes (encoding miRNAs) are transcribed as independent transcriptional units by RNA polymerase II (PolII) and may be regulated through *cis* regulatory promoter elements [9, 19–21]. Mature miRNAs are processed from longer primary transcripts (pri-miRNAs) and cleaved by a core microprocessor complex consisting of type III RNAse, DICER-LIKE1 (DCL1), zinc finger protein SERRATE (SE), and dsRNA binding protein HYPONASTIC LEAVES1 (HYL1) [22–24]. Stepwise cleavage generates precursor miRNAs (pre-miRNAs) at the first step and mature miRNAs in the second cleavage reaction step. miRNAs are then transported to the cytoplasm and loaded onto ARGONAUTE proteins to form the sequence-specific RNA-induced silencing complex (RISC) [25]. Once programmed with an miRNA, RISC can silence target genes by translational inhibition, mRNA cleavage, or heterochromatin formation [26]. As trans-acting regulators, foremost efforts were put into elucidating the temporal and spatial expression changes of individual miRNAs and targeted genes, while the exact regulatory mechanism is often obscure. Therefore, transcriptional and posttranscriptional control of miRNA expression is the subject of intensive research, yet there is a knowledge gap with respect to its crosstalk with chloroplast-derived signals [27–29].

Light propels photosynthesis, which is a well-established source of reactive oxygen species (ROS) in plants [30]. Perturbations in photosynthesis during light stress conditions lead to intensified production of ROS—^1^O_2_ (singlet oxygen) at PSII and H_2_O_2_ and O_2_^•−^ at PSI. To date, only a few ^1^O_2_-dependent retrograde signaling pathways have been reported. In the grana margins (GM), ^1^O_2_ is mostly produced from tetrapyrrole biosynthesis intermediates and activates three signaling pathways: 1) EX1/EX2-dependent programmed cell death, 2) E3 ubiquitin ligase plant U-box 4 (PUB4)-dependent selective chloroplast degradation, and 3) a newly described SAFEGUARD1 (SAFE1)-dependent pathway [31–33]. Nevertheless, ^1^O_2_ is also produced in the grana core (GC), and the signal is transduced through the 1) β-cyclocitral (β-CC)-dependent or 2) OXI1 kinase-mediated signaling pathways [34, 35].

In addition to ROS production, electron flux between PSII and PSI also results in the redox regulation of photosynthetic electron transport chain (PET) components. Accordingly, HL causes plastoquinone (PQ) reduction, located downstream of PSII, to plastoquinol (PQH2). These PQ/PQH2 redox state changes are responsible for regulating at least 750 nuclear genes [3, 36]. In summary, the PQ pool redox state and ^1^O_2_-related signals may contribute to retrograde communication because they originate in chloroplasts and induce nuclear gene expression changes during HL conditions. There is some evidence for a PQ pool oxidation/reduction role in miRNA processing [37], as well as an influence on alternative splicing [38]. Nevertheless, the coupling of ^1^O_2_ signaling with miRNA abundance is currently an unexplored part of miRNA and retrograde signaling cross-talk.

Here, we demonstrate that HL causes changes in the miRNA levels in *A. thaliana* rosettes. The possible role of retrograde signals, especially ^1^O_2,_ in miRNA expression was studied to verify the putative link to specific ^1^O_2_ signaling pathways. To achieve that, analyses of an *A. thaliana flu* mutant*,* conditionally producing ^1^O_2_, and mutants impaired in ^1^O_2_-mediated retrograde communications exposed to HL were conducted. β-CC application, a volatile retrograde signaling mediator, followed by monitoring of pri-miRNA expression, was also implemented. Taken together, our study is the first report linking ROS production and miRNA expression in the context of light stress. Nevertheless, future work should be undertaken to fully understand this mechanism and its biological function.

## Results

### High light induces miRNA expression changes

**Fig. 1:**
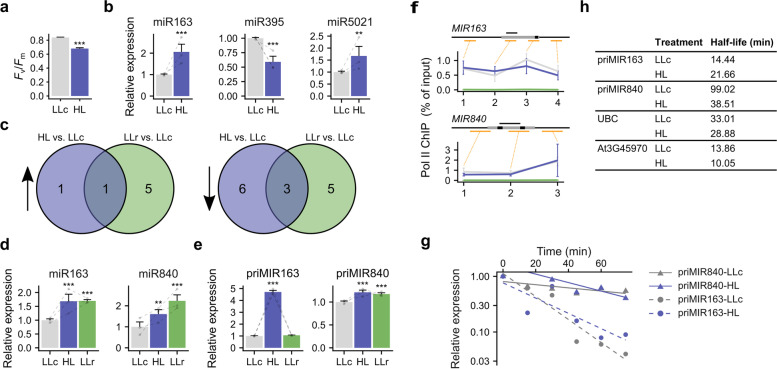
HL induces miRNA expression changes in *Arabidopsis thaliana* shoots. **a** *F*_v_/*F*_m_ measured in 4-week-old Arabidopsis plants grown in LL under hydroponic conditions. LLc – control plants; HL—plants exposed to HL for 2 h (n = 18). **b** TT-qRT PCR for selected miRNA-targeted transcripts of nuclear encoded proteins localized in chloroplasts under LLc and HL. Transcript levels were normalized with respect to small nucleolar85 RNA (sno85) and small nucleolar101 RNA (sno101). **c** Results of a microtranscriptomic screening for miRNA expression changes in LLc, HL and LLr – plants exposed to HL for 2 h and a subsequent recovery for 4 h in LL. **d** TT-qRT PCR for miR163 and miR840 and **e** qRT-PCR for pri-miR163 and pri-miR840 in Col-0 LLc, HL and LLr plants. Transcript levels were normalized with respect to sno85 and sno101 (for miRNAs) or the *PP2A* and *UPL7* genes (for pri-miRNAs), respectively. **f** Occupancy of PolII on *MIR* genes. Line charts present ChIP profile of total PolII on examined genes. Grey lines represent results for LLc plants and blue lines represent results for HL plants. Above each chart, gene structure is shown with black boxes representing miR, and grey boxes representing primary transcripts (pri-miRs).Orange lines show amplified regions (primer localization used for qRT-PCR analysis). Above each gene structure 100 bp scale is shown. **g** RNA stability assay performed on Arabidopsis seedlings in control plants (LLc) and plant exposed to HL (HL). Degradation curves after cordycepin treatment (plot) were used to calculate half-life of pri-miR840, pri-miR163, UBC (control) transcripts and short-lived mRNA transcribed from gene At3G45970 (table **h**). Presented values are averages from three biological replicates. For better clarity of chart only pri-miRNAs data were presented. Asterisks indicate significant differences according to Tukey’s HSD test (panel: a,b,d,e) or t-test (panel f) at the level of ** ≤ 0.01 and *** ≤ 0.001. Mean values ± SDs (*n* = 3) were provided

To assess the impact of HL on miRNA expression changes, low light (LL)-acclimated *Arabidopsis thaliana* plants grown in hydroponic conditions were subjected to HL treatment for 2 h (HL), which was 10 times greater than that under the LL growth conditions (see section Methods). Such HL treatment was shown to cause a photoinhibitory effect and promote chloroplast to nucleus signaling [39–41]. The photoinhibitory effect was confirmed by measuring PSII activity, which was determined as the maximum quantum efficiency of PSII expressed as the *F*_v_/*F*_m_ parameter, the ratio of variable to maximum chlorophyll fluorescence (Fig. 1a). Additionally, the induction of *APX2, ELIP1*, and *RRTF1*, known as HL response marker genes [1, 42, 43], confirmed the stress effect of the HL treatment (Additional file 1: Fig. S1). Since we hypothesized that miRNAs are involved in light-triggered gene regulation, we first searched the list of *Arabidopsis* nuclear genes coding for chloroplast proteins using available small RNA target prediction software and tested several miRNA candidates [44, 45] (Fig. 1b; Additional file 1: Table. S1). The expression of miR163 and miR5021 was upregulated in plants treated with HL (HL) versus that in control plants (LLc) with a simultaneous decrease in miR395 expression (Fig. 1b). To identify other miRNAs connected with the HL response, we performed microtranscriptomic sequencing of LLc-, HL- and LLr (LLr—HL-treated plants followed by 4 h recovery in LL)-treated plants, which allowed us to identify 21 miRNA candidates regulated by HL. Of these, 7 were up- and 14 were downregulated, with confirmed upregulation of miR163 under HL (Fig. 1c; Additional file 1: Fig. S2; Fig. S3). The observed HL-triggered miRNA expression changes were limited, dynamic, and rather subtle (fold changes ranging from approximately 0.4 to 2.8). Selected candidates were validated by the two-tailed RT-qPCR method (TT-RT qPCR), [46] and two of them, miR163 and miR840, which were upregulated just after HL stress (in the microtranscriptomic screen), were chosen for further analysis (Additional file 1: Fig.S4; Fig S5; Fig. 1c, d). HL induction was also visible 4 h after HL stimuli (LLr), although only in the case of miR840 it was confirmed by both RNAseq and qPCR analysis (Additional file 1: Fig. S2; Fig. S4; Fig. 1d). The involvement of miR163 and miR840 in the HL response is not surprising because miR163 was previously found to be induced by light during seedling de-etiolation [47, 48] after 6 h of HL [12] or red light treatment [49], and miR840 was described as a gamma-ray-responsive miRNA [16].

The maturation of functional miRNAs is a multistep process; consequently, a better understanding of their regulation cannot be limited to the analysis of mature forms of miRNAs. Therefore, we also monitored the expression of pri-miR163 and pri-miR840 (Fig. 1e). Pri-miR163 was highly accumulated under HL, while the expression of pri-miR840 was slightly increased under both HL and LLr. Although the expression of pri-miR163 is elevated 5 times under HL, changes in miR163 level do not exceed twofold (Fig. 1d) On the other hand, miR840 exhibits comparable level of expression fold changes at the analyzed stages of miRNA biogenesis. This divergence between miR163 and miR840 may be caused by different maturation process of analyzed miRNAs. It was proven that physical interactions between the DCL1 and HYL1 proteins are necessary for precise miR163 precursor processing [23]. Generally, most miRNAs require HYL1 for their processing; therefore, they are defined as HYL1-dependent miRNAs, and their precursors overaccumulate in *hyl1* mutant plants. Nevertheless, HYL1 activity is not crucial for the maturation of all miRNAs [9, 23, 50, 51]. Moreover, several miRNAs may become HYL1/SE conditionally independent at decreased temperatures [52]. Therefore we investigated pri-miRNA levels in the Col-0 and *hyl1* mutant (Additional file 1: Fig. S6). Pri-miR163 overaccumulated in the *hyl1* mutant, while the pri-miR840 level was similar in Col-0 plants, which clearly indicated that pri-miR163 maturation is HYL1-dependent (Additional file 1: Fig. S6). Remarkably, the mature miR840 level in *hyl1* was several-fold greater than that in Col-0 (Additional file 1: Fig. S6). Since the level of pri-miRNAs is an outcome of the transcription rate and stability of transcript we performed PolII-ChIP assay (Fig. 1f). This experiment show that no statistical differences in PolII occupancy was detected at tested miRNA genes between LL and HL conditions. On the other hand, GUS staining revealed that HL enhanced pri-miR163 expression (Additional file 1: Fig. S7). Probably, observed changes are connected with regulation of pri-miRNA stability rather than the transcription rate or PolII-ChIP experimental setup missed shorter gene activation periods. To test first hypothesis cordycepin assay was carried out. Stability of pri-miR163 increases after HL treatment in opposite to pri-miR840 whose half-life is substantially reduced (Fig. 1g-h).

In summary, miRNA sequencing followed by RT-qPCR analysis confirmed the miRNA response to HL. Moreover, this response was regulated at the different stages of miRNA biogenesis.

## The role of PQ redox status in miRNA expression is not conclusive

**Fig. 2:**
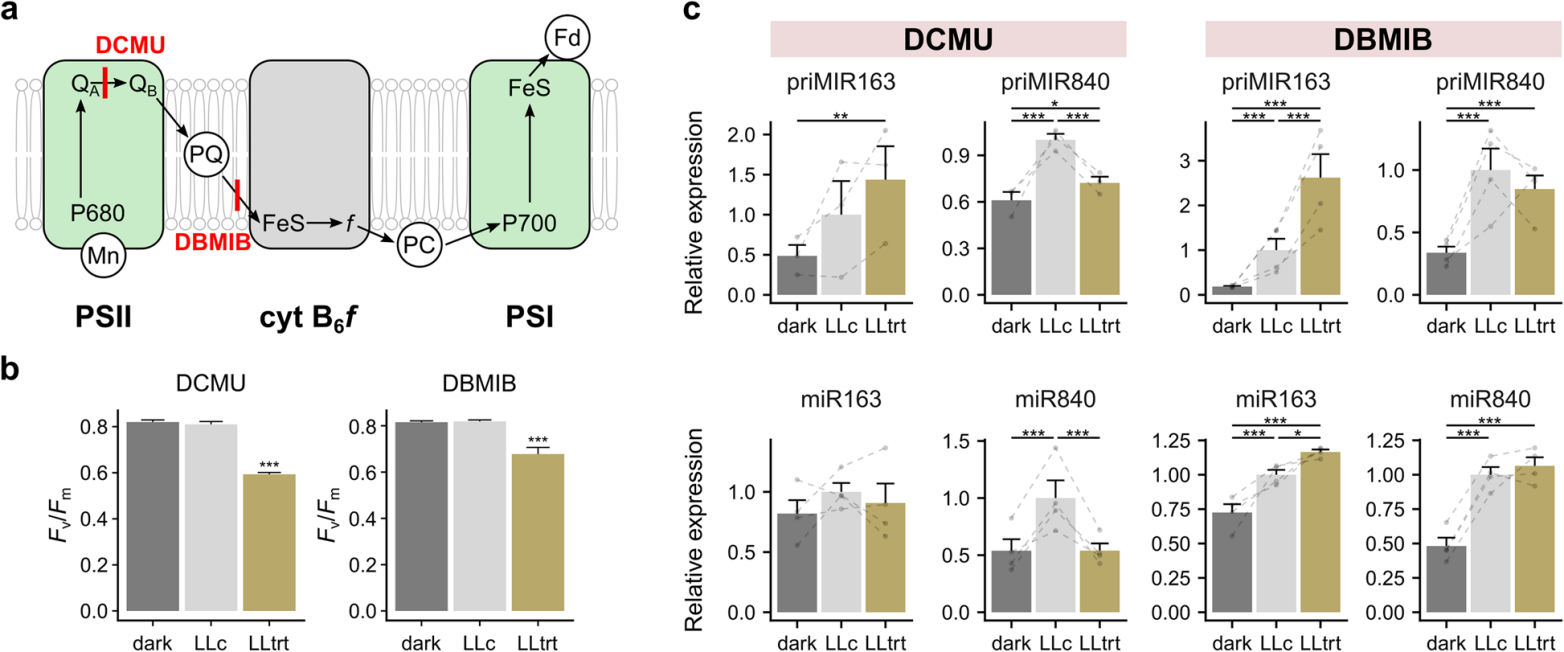
The role of PQ redox status in miRNA expression. **a** Scheme representing the action of DCMU and DBMIB in the photosynthetic electron transport chain. **b** Treatment with DCMU and DBMIB influences the maximum efficiency of PSII expressed as the Fv/Fm parameter. Plant material: dark—plants kept in darkness for 4 h; LLc—control plants in LL; LLtrt—plants treated with DCMU or DBMIB for 4 h and kept in LL. Asterisks indicate significant differences according to Tukey’s HSD test at the level of *** ≤ 0.001. Mean values ± SDs (*n* = 9) were provided. **c** qRT-PCR for pri-miR163 and pri-miR840 after using DCMU or DBMIB (upper panel). Transcript levels were normalized with respect to the *PP2A* and *UPL7* genes. TT-qRT PCR for miR163 and miR840 after treatment with DCMU or DBMIB (bottom panel). Transcript levels were normalized with respect to sno85 and sno101. Asterisks indicate significant differences according to Tukey’s HSD test at the level of * ≤ 0.05, ** ≤ 0.01 and *** ≤ 0.001. Mean values ± SDs (n = 3) were provided

To test the engagement of chloroplast-derived signals, we used inhibitors of PET because its components act as excess light messengers and nuclear gene expression regulators [1, 53–55]. The PQ redox status can be easily modulated using PET inhibitors. DCMU (3-3,4-dichlorophenyl-1,1-dimethylurea) blocks the PSII PQ binding site, oxidizing the PQ pool [56]. DBMIB (2,5-dibromo-6-isopropyl-3-methyl-1,4-benzoquinone) has been used as a specific inhibitor of plastoquinol oxidation at the Qo binding site of cytochrome b6f, causing reduction of the PQ pool [57] (Fig. 2a). Plants were kept in the dark (dark) or LL (LLc), and plants were treated with DCMU or DBMIB for 4 h in LL (LLtrt) (Fig. 2b,c; Additional file 1: Fig. S8). The effective inhibitor concentrations were determined by monitoring the *F*_v_/*F*_m_ parameter, which decreased 4 h after chemical application (Fig. 2b). Since we showed that miRNAs can be regulated at different biogenesis stages, pri-miRNAs and mature miRNAs were monitored. Pri-miR163 and pri-miR840 were upregulated in light-treated plants compared to dark-treated plants (Fig. 2c). DCMU further enhanced pri-miR163 induction, whereas light-dependent pri-miR840 induction was almost completely abolished. In the DBMIB experiment, pri-miR163 was strongly upregulated, while the pri-miR840 level was downregulated compared to that in the LL control plants but remained significantly elevated compared to that in dark-treated plants. Since the dominant effect was related to the dark–light switch, we deduce that the increase in pri-miRNAs may not be dependent on PQ because it occurred in the DCMU treatment (PQ oxidized) as well as DBMIB treatment (PQ reduced). However, in the case of pri-miR840, DCMU seemed to block the effect of light to some extent (similar results were also observed in pri-miR319b, which was upregulated in the LLr treatment in the microtranscriptomic screening (Additional file 1: Fig. S2; S9). Since the analysis with inhibitors was not conclusive, we decided to check the expression level of pri-miR163 and pri-miR840 in protein kinase STATE TRANSITION 7 (*stn7*) and SALICYLIC ACID INDUCTION DEFICIENT 2 protein (*sid2*) mutant plants, which were previously shown to have a reduced PQ level compared to that of Col-0 plants (Additional file 1: Fig. S10) [58]. We observed no differences in pri-miRNA levels (and miRNA) in the tested mutants, which suggests that PQ redox status is not responsible for changes in pri-miRNAs.

Next, we tested the abundance of mature miRNAs after treatment with DCMU and DBMIB. Surprisingly, changes exhibited in pri-miRNAs were largely abolished in the mature forms (Fig. 2c). Although we still observed statistically significant differences in the miR163 expression level, the magnitude of the presented changes was scaled down (Fig. 2c, DBMIB panel) or abolished completely (Fig. 2c, DCMU panel). Because pri-miR163 induction in the DBMIB treatment was greater than that in DCMU, it can be assumed that the miRNA level response was almost equally reduced in both treatments. Simultaneously, the ratio and pattern of miR840 changes were maintained from the pri- to mature miRNAs, indicating different miR163 and miR840 maturation processes.

In summary, the presented results are not conclusive in defining the role of the PQ pool redox state in the light-triggered regulation of miRNAs. Thus, the impact of other possible retrograde signals should be considered.

## Regulation of pri-miRNA expression is not dependent on EX1-dependent ^1^O_2_ signaling

Pri-miRNA expression changes induced by light were similar for both the DCMU and DBMIB treatments, which suggests that transcription of miRNAs may be induced upstream from PQ. The PSII light-harvesting antenna complex is the main place where the production of highly reactive ^1^O_2_, a photosynthesis byproduct, occurs [59–63]. Under mild stress conditions, ^1^O_2_ may promote programmed cell death by activating two nucleus-encoded proteins, EXECUTER1 (EX1) and EX2, which are located in the chloroplast GM, where chlorophyll is synthesized and the PSII repair cycle takes place [31, 64–67]. Under light stress, various ROS can be generated simultaneously; therefore, it is impossible to analyze the specific biological activity of ^1^O_2_. Fortunately, a *flu* conditional mutant that selectively overproduces ^1^O_2_ from the photosensitizer protochlorophyllide (Pchlide) initiated numerous studies on the signaling functions of this ROS [31, 68, 69]. The *flu* mutant in continuous light displays the wild-type phenotype because in these conditions, Pchlide is immediately photoreduced to chlide and consequently does not reach the level necessary for elevated ^1^O_2_ production [70]. By transferring light-grown *flu* plants to darkness for a period and then re-exposing them to light, we can easily modulate the Pchlide level because it accumulates proportionally over time and consequently produces ^1^O_2_. Interestingly, these symptoms are abrogated in *flu/ex1* double mutants, demonstrating that Pchlide accumulation is not enough to trigger ^1^O_2_ signaling and requires EX1 protein function [67, 70, 71].

**Fig. 3:**
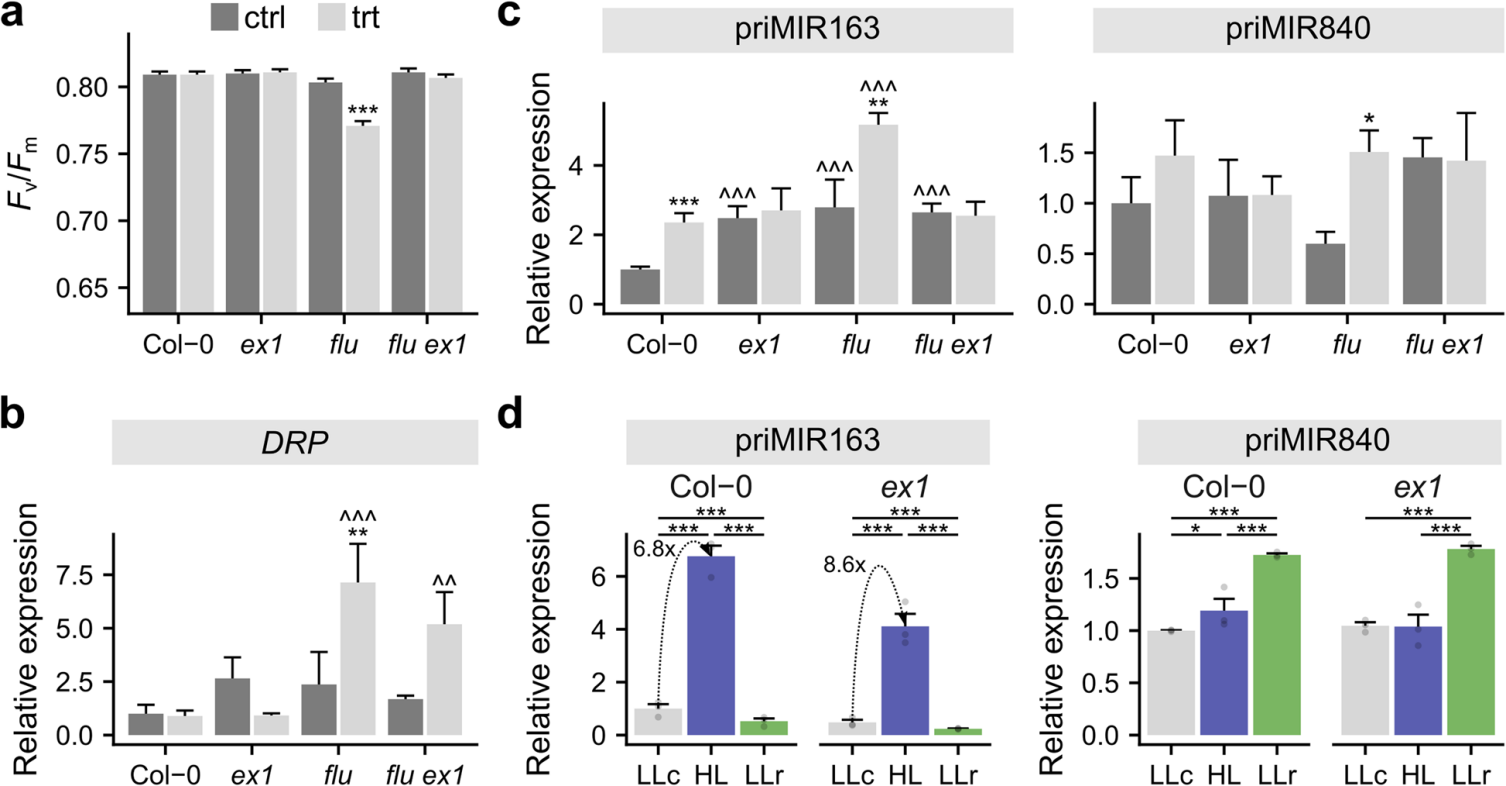
Regulation of pri-miRNA expression is not dependent on EX1-dependent ^1^O_2_ signaling. **a** The *F*_v_/*F*_m_ parameter measured in control (ctrl) plants (grown in constant light (CL)) and treated (trt) plants (plants grown for 2 weeks in CL, placed for 12 h in darkness, and re-exposed for 2 h to LL) of the Col-0, *ex1*, *flu*, and *flu*/*ex1* genotypes (n = 9–10). **b** qRT-PCR of the *DRP* gene in the Col-0, *ex1*, *flu*, and *flu*/*ex1* genotypes in ctrl and trt plants. **c** qRT-PCR for pri-miR163 and pri-miR840 in the Col-0, *ex1*, *flu*, and *flu*/*ex1* genotypes in ctrl and trt plants. **d** qRT-PCR for pri-miR163 and pri-miR840 in Col-0 and *ex1* plants LLc-control plants; HL-plants exposed to HL for 2 h; LLr-plants exposed to HL for 2 h and subsequent recovery in LL for 4 h. Transcript levels were normalized with respect to the *PP2A* and *UPL7* genes. Asterisks indicate significant differences according to Tukey’s HSD test at the level of * ≤ 0.05, ** ≤ 0.01 and *** ≤ 0.001. In (b) and (c), * indicates significance within the same genotype, while ^ indicates comparison to Col-0 within the same conditions. Mean values ± SDs (*n* = 3) were provided

The potential role of ^1^O_2_ in the induction of *MIR* expression was verified by analysis of pri-miR163 and pri-miR840 expression changes in wild-type Col-0 and *flu*, *ex1*, and *flu/ex1* double mutant seedlings grown in LL for 2 weeks and transferred to darkness for 12 h, then subsequently returned to LL for 2 h for ^1^O_2_ generation. After that time, *F*_v_/*F*_m_ was measured in treated (trt) and control (ctrl, kept continuously in LL) plants (Fig. 3a). Treated *flu* seedlings exhibited a stress response (decrease in the *F*_v_/*F*_m_ value) because of ^1^O_2_ overproduction in PSII. This decline in photosynthetic parameters was not exhibited in Col-0, *ex1*, and *flu/ex1* plants, which implies that ^1^O_2-_mediated and EX1-dependent signaling takes place under noninhibitory light and that ^1^O_2_ produced in *flu* background seedlings does not directly damage PSII [67]. Moreover, ^1^O_2_ release was shown by elevated *DRP* expression, a known ^1^O_2_ marker gene [72, 73], in *flu* background plants (Fig. 3b). Pri-miRNA163 and pri-miR840 were upregulated in *flu* (pri-miR840 changes were statistically significant); however, this elevated level was also observed in Col-0, suggesting that it is not connected with EX1-dependent ^1^O_2_ signaling. Although Pchlide is first synthesized in the GM, when the darkness time exceeds 8 h (in the case of the *flu* and *flu/ex1* plants), after reillumination, Pchlide also accumulates in GC and slightly accumulates in stroma lamellae [32]. Thus, the 12 h of darkness used in our experiments may also activate other EX1-independent ^1^O_2_ signaling pathways, which would explain the greater pri-miR840 level. No changes in pri-miRNAs in *flu/ex1* plants indicate that regulation of these miRNAs is not dependent on EX1 (Fig. 3c). To confirm these observations, we exposed the *ex1* mutant to HL stress for 2 h, and subsequent analysis of the pri-miRNA expression levels was carried out (Fig. 3d). Pri-miR163 expression was induced by HL in both Col-0 and *ex1* plants. Although the pri-miR163 expression level in HL-treated mutant plants was reduced, it was already reduced in *ex1* control plants, and the fold changes for *ex1* were even greater than those in Col-0 (8.6 in *ex1* versus 6.8 in wild-type plants) (Fig. 3d; Additional file 1: Fig. S11). We observed a slight increase in pri-miR840 expression in HL-treated Col-0 plants, while expression changes in LLr plants were nearly identical for Col-0 and *ex1*. Thus, our results suggest that the EX1-^1^O_2_ signaling pathway is not engaged in HL-induced miRNA regulation.

## pri-miRNA expression is regulated by β-CC-dependent ^1^O_2_ signaling

**Fig. 4:**
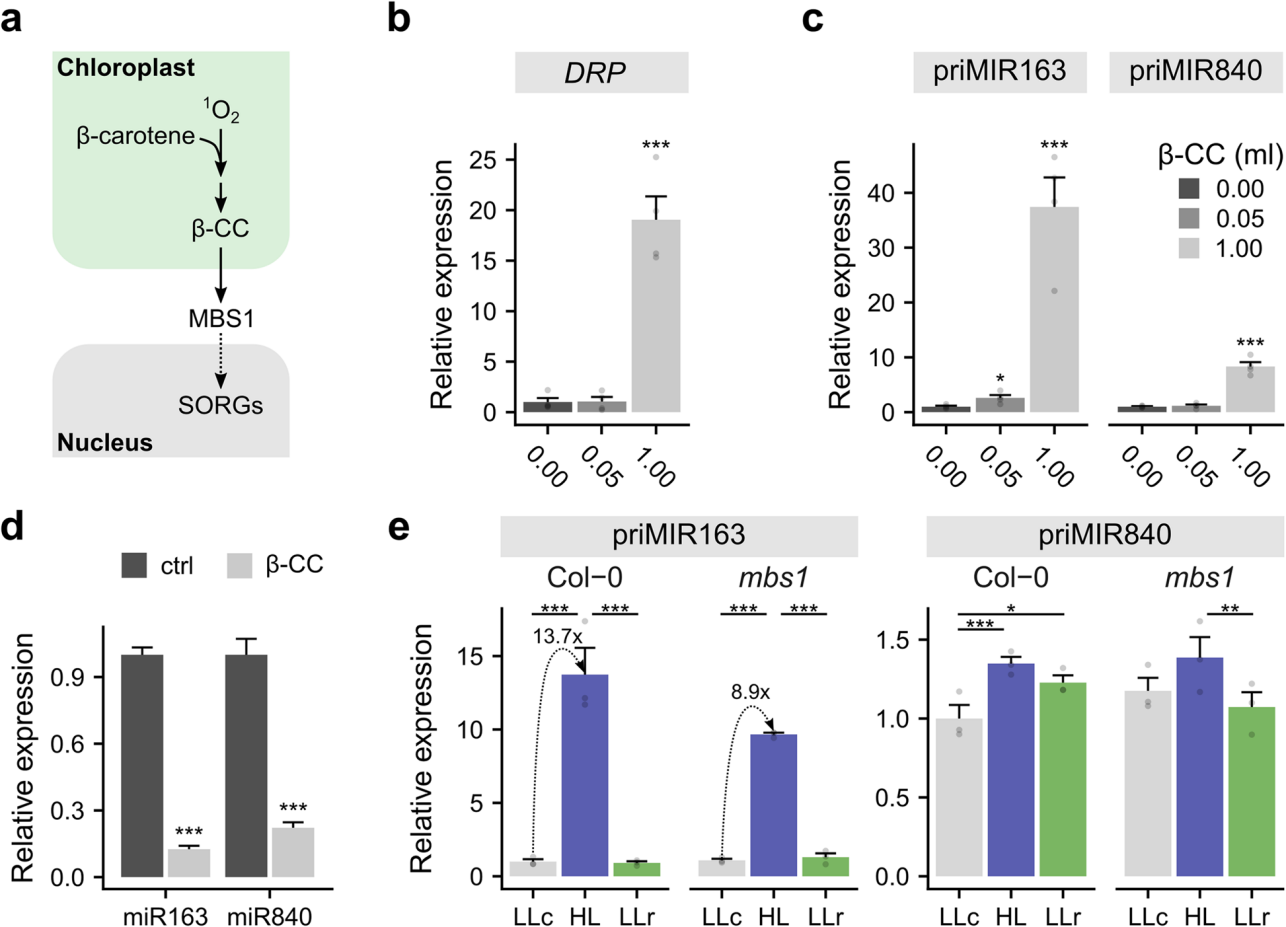
Regulation of pri-miRNA by β-CC-dependent ^1^O_2_ signaling. **a** Scheme represents the β-CC-dependent ^1^O_2_ signaling pathway induced in HL conditions. β-CC is formed in PSII as a result of β-carotene oxidative breakdown under HL conditions. MBS1 is a downstream component that transduces stress information to the nucleus, where it influences the expression of singlet oxygen responsive genes (SORGs). **b** qRT-PCR for the *DRP* gene and **c** pri-miR163 and pri-miR840 in control plants (0.00) and after β-CC treatment (0.05 ml and 1.00 ml) for 4 h. **d** TT-qRT PCR for miR163 and miR840 level in plants treated by β-cc. ctrl- control plants, trt—plants treated with 1.0 ml of β-CC. Transcript levels were normalized with respect to sno85 and sno101. **e** qRT-PCR for pri-miR163 and pri-miR840 in Col-0 and *mbs1* plants. LLc-control plants; HL-plants exposed to HL for 2 h; LLr-plants exposed to HL for 2 h and subsequent recovery in LL for 4 h. Transcript levels were normalized with respect to the *PP2A* and *UPL7* genes. Asterisks indicate significant differences according to the Tukey HSD test at the level of * ≤ 0.05, ** ≤ 0.01, *** ≤ 0.001. Mean values ± SDs (n = 3), were provided

^1^O_2_ formation may also activate EX1-independent signaling, which occurs in photoinhibitory conditions. In such circumstances, light stress leading to β-carotene oxidative breakdown results in the release of small volatile compounds such as β-CC, which is known to induce ^1^O_2_-responsive genes (Singlet Oxygen Responsive Genes—SORGs, Fig. 4a) [34, 64]. Therefore, we tested the possible role of the β-CC-^1^O_2_-dependent pathway in miRNA expression. β-CC treatment (for details see Methods section) was utilized, and then we examined the *DRP* expression level to confirm that ^1^O_2_ signaling was activated (Fig. 4a-b). Significantly increased *DRP* expression was detected after β-CC (1 ml) application (Fig. 4b). Subsequently, we analyzed pri-miR163 and pri-miR840 expression levels in the same experimental setup. Pri-miR163 abundance was elevated at both β-CC doses (i.e., 50 µl and 1 ml), whereas pri-miR840 abundance was elevated at the greater dose only (Fig. 4c). Surprisingly, the level of both miRNAs decreased drastically after application of 1 ml of this chemical (Fig. 4d). Next, we considered the possible role of this retrograde signaling pathway in miRNA regulation. The β-CC signaling pathway requires the MBS1 protein, which is positioned downstream of β-CC, to mediate ^1^O_2_ signal transfer to the nucleus [68]. MBS1 was identified in a *Chlamydomonas reinhardtii* (green alga) mutant genetic screen displaying a defect in response to ^1^O_2_ generated by the photosensitizer methylene blue [74]. Moreover, an *A. thaliana mbs1* mutant and *mbs1*/RNAi-*mbs2* double mutants were shown to be more susceptible to HL conditions. Thus, while in wild-type plants β-CC causes induction of SORGs, resulting in greater photooxidative stress tolerance [34], the insertional knockdown *mbs1* mutant exhibits deregulation in SORG expression after β-CC treatment and consequently does not achieve β-CC-induced HL tolerance [75]. In line with this idea, we exposed wild-type and *mbs1* mutant plants to an episode of HL, and then we analyzed pri-miRNA expression levels (Fig. 4e; Additional file 1: Fig. S12). HL induces the expression of both pri-miR163 and pri-miR840 in Col-0, and the scale of this induction is much greater in pri-miR163. This response was noticeably reduced in *mbs1* plants for both pri-miRNAs (Fig. 4e). The pri-miR163 induction was almost 40% weaker (13.7- versus 8.9-fold changes for Col-0 and mbs1, respectively), whereas pri-miR840 induction lost statistical significance in mutant plants (Fig. 4e). However, when we compared these data within the treatment, not within the genotype, we still observed a significant reduction in the expression level for pri-miR163 but not for pri-miR840 in HL (Additional file 1: Fig. S12). Only a partial effect of the *mbs1* mutation on pri-miRNA induction suggests the existence of other parallel regulatory mechanisms or, more likely, the redundant role of a close MBS1 homolog – MBS2 [75]. Thus, chemical (β-CC) and partial genetic premises (*mbs1* study) imply the potential role of ^1^O_2_ in the regulation of miRNA expression.

## Discussion

### Light affects posttranscriptional gene regulation

The ability to transfer stress signals from chloroplasts to the nucleus and consequently influence nuclear gene expression is indispensable for plant adaptive strategies. Thus, effective retrograde communication and its linkages with RNA metabolism have been extensively studied [76]. For example, it was demonstrated that changes in the PQ redox pool may regulate the alternative splicing process [38, 77, 78]. Recently, the basis of these mechanisms was revealed, showing that light increases the PolII elongation rate, while in darkness, elongation is reduced, which is consistent with the kinetic coupling model also found in mammalian cells [79, 80]. Light also influences other posttranscriptional gene expression regulation mechanisms, including the regulation of miRNAs [81, 82].

## HL reveals incosistency in pri-miRNA and miRNA abundance

Light may affect miRNA expression at different stages of biogenesis. For example, light has a negative impact on miRNA processing (described as the phenomenon of inconsistency in miRNA levels) and is manifested by a reduction in miRNA response compared to significantly greater changes in pri-miRNA abundance, which was observed in the context of de-etiolation [83]. This light-dependent mechanism decreases the capability of miRNA processing, and activity of SMALL RNA DEGRADING NUCLEASE 1 (SDN1), which reduces the half-life of several miRNAs in de-etiolated seedlings, conferring relatively constant miRNA levels [83]. Recently, it was demonstrated that the forkhead-associated domain 2 (FHA2) protein is the light-stabilized suppressor of miRNAs through interactions with DCL1 and HYL1 [84, 85]. Briefly, FHA2 inhibits DCL1 enzyme activity and facilitates the accessibility of HYL1 to pri-miRNAs. Despite all of these reports, there is a knowledge gap concerning the role of light stress in miRNA processing, and the majority of current observations are based on nonstressed conditions. Certainly, there is some kind of negative regulation of miRNA processing during HL because we also observed inconsistency in miRNA abundance after HL treatment. For instance, upon HL, the fold changes for pri-miR163 ranged from 5 to 15 in Col-0 (Fig. 1e, Fig. 3d, Fig. 4e). Simultaneously, the changes of the mature miRNA did not exceed twofold, regardless of the experimental setup or methodology used (Fig. 1b,d, Additional file 1: Fig. S4). These results are compatible with those of experiments that used PET inhibitors for pri-miR163 (Fig. 2). Conversely, in the case of pri-miR840, mature miRNA changes were almost identical to those at the pri-miRNA level.

## Functioning of the core components of miRNA biogenesis is disturbed by HL

We concluded that the discrepancy between the level of pri-miRNAs and their mature forms may depend on HYL1 activity because we observed accumulation of primary transcripts in the case of pri-miR163 in the *hyl1* mutant but not for pri-miR840 (Additional file 1: Fig. S6). Interestingly, the mature miR840 level in *hyl1* was several-fold greater than that in Col-0, indicating that miR840 biogenesis was more efficient in the absence of HYL1 (in the middle of the photoperiod light phase). This highlights the putative role of HYL1 as the microprocessor component underlying the regulation of miRNA expression by light. Since light may affect HYL1 localization and activity [37], such a conclusion seems justifiable. Nevertheless, there are some contradictory reports referring to light-dependent HYL1 regulation. For example, during the dark to red light transition, phytochrome-interacting factor 4 (PIF4) destabilized HYL1 and DCL1 [82, 86]. However, COP1 (constitutive photomorphogenic 1), a RING-finger E3 ligase and negative photomorphogenesis regulator, contributed to light-dependent proteolytic stabilization of the HYL1 protein during the daytime [87]. Additionally, HYL1 activity was maintained by its nucleo-cytoplasmic relocation and phosphorylation/dephosphorylation changes [82, 88, 89]. The nuclear pool of HYL1 in prolonged darkness is phosphorylated, which protects it from degradation. After plant reillumination, HYL1 is dephosphorylated to rapidly restore miRNA production [37]. In addition, the possible influence of chloroplast signals with respect to HYL1 posttranscriptional regulation was suggested because DCMU almost completely blocks HYL1 dephosphorylation after prolonged darkness [37]. Although there is some evidence of light-triggered HYL1 regulation, there is a lack of data about HL-governed changes in microprocessor functioning. Moreover, in addition to the importance of HYL1, DCL1 plays a dominant role in miRNA processing. Like HYL1, DCL1 is subject to multifaceted regulation [29]. For example, it was demonstrated that DCL1 highly accumulates in de-etiolation, but the processing activity decreases; consequently, the level of mature miRNAs is nearly unaltered [83]. Although we observed no changes in DCL1 accumulation after HL exposure (Additional file 1: Fig. S13), its activity is likely disturbed by this stress. This suspicion was supported by a dramatic decrease in miRNA levels after β-CC treatment (Fig. 4d). One of the plausible explanations is the negative regulation of microprocessors by overproduced ROS (major engagement of ^1^O_2_). Probably the applied dose of β-CC causes not only stronger pri-miRNAs induction but at the same time has much more severe impact on further steps of miRNAs biogenesis than HL. The exact mechanism by which HL stress adjusts the functionality of microprocessor components to suppress miRNA biogenesis still remains unknown. Further study on the role of retrograde signals may contribute to a deeper understanding of HL-regulated miRNA expression changes.

## Retrograde ^1^O_2,_ signaling is important in miRNA regulation

To date, knowledge about the role of plastid-derived signals in miRNA regulation in the context of light signaling, in addition to the possible role of DCMU in changing the HYL1 phosphorylation status, is still vague and limited to a few but significant reports. For example, the involvement of the plastid-nucleus located DNA/RNA binding protein WHIRLY1 in miRNA expression regulation during light stress in barley was postulated [90]. sRNA sequencing combined with mRNA/lncRNA sequencing on Arabidopsis wild-type plants and two retrograde signaling mutants, *gun1* and *gun5,* treated with the herbicide norflurazon shed light on cross-talk between sRNAs (including miRNAs) and retrograde signaling [91]. Additionally, the functional role of chloroplast-derived signals in miRNA regulation was postulated recently by Fang et al., 2019 [92], in the context of heat stress. They showed that tocopherols enhance the accumulation of 3’-phosphoadenosine 5’ phosphate (PAP), a retrograde nuclear exonuclease 2 (XRN2) inhibitor. Thus, tocopherols and PAPs are positive regulators of miRNA biogenesis because their accumulation represses XRN2, which negatively regulates mRNA and pri-miRNA levels by the degradation of uncapped 5’ mRNA. Since tocopherols (together with carotenoids) are reported to protect PSII against photoinhibition and lipid peroxidation by ROS, questions about the role of oxidative stress (including ^1^O_2_) in this regulation have been raised [61, 93, 94]. This is especially important because PAP accumulates during drought- and HL-induced oxidative stress, and its level is regulated by the SAL1 enzyme, which dephosphorylates PAP to AMP [95]. Interestingly, the *alx8* mutant, which accumulates PAP, has a significantly higher level of PXMT1, which encodes a 1,7-paraxanthine methyltransferase, a target gene of miR163 [95]. Since PAP effectively suppresses not only nuclear XRN2 and XRN3 but also cytoplasmic XRN4, which functions in the degradation of miRNA target cleavage products, this result is reasonable [96]. In addition, *alx8* also exhibited an increase in the pri-miR163 level, with a simultaneous decrease in mature miRNA forms (Aditional file 1: Fig. S14).Considering that the increased pri-miRNA level in HL can be controlled not only by inhibition of nuclear XRNs but also by a higher pri-miRNA transcription rate, we performed PolII-ChIP analysis. There were no changes in PolII occupancy at the tested *MIR* genes between LL and HL treatments, arguing against the second possibility (Fig. 1f). However, promoter fusion with the beta-glucoronidase gene showed enhanced transcription of pri-miR163 in HL (Additional file 1: Fig. S7). For miR163, there is a report that HY5 regulates light-responsive transcription of miR163 [97]. The lack of changes in PolII occupancy seem to contradict these results; however, PolII-ChIP measures the transcription rate as a function of PolII occupancy at the time of analysis – 2 h of HL. Nonetheless, there is the possibility that the transcription rate for miR163 and miR840 is in fact changed at the beginning of HL treatment because pri-miRNA expression of those miRNAs was even higher in plants exposed to HL for 30 min (Additional file 1: Fig. S15). Since the DRP level was also more abundant after 30 min of treatment, we consequently considered ^1^O_2_ to be a putative signal that induces miRNA expression. Notably, the effective transcription rate can vary even when PolII occupancy at tested genes is stable because PolII can pause or even stall [98]. Perhaps the pri-miRNA level is determined by the interplay between pri-miRNA transcription and stability; therefore, we also verified the half-life of these pri-miRNAs using a cordycepin assay (Fig. 1g-h). The obtained results suggest that pri-miR163 is more stable in HL, while the stability of pri-miR840 is reduced (Fig. 1g-h). The differences between pri-miR163 and pri-miR840 degradation in HL consistently indicate that HYL1 may be an important determinant of the observed changes. It is possible that HL causes an increase in the transcription of some miRNAs and simultaneously disturbs the activity of HYL1 and DCL1. Collectively, our results suggest that changes in pri-miRNA levels are tuned by changes in pri-miRNA transcription and stability. Although we present the link between miRNA expression and ROS (i.e., ^1^O_2_) signaling upon HL stress, the involvement of other retrograde signals remains to be investigated, especially in the context of miRNA biogenesis regulation. Further investigation of these aspects will lead to a better understanding of how miRNA expression and processing machinery cooperate upon HL. Such research should include the biological function of miRNA targets and their evolutionary context.

## Conclusions

In summary, we provided several lines of evidence that pinpoint the function of ^1^O_2_ in the regulation of miRNA expression during HL conditions. The negative impact of high light intensity on some mature miRNA levels and simultaneous accumulation of their primary transcripts was also observed. We postulate that microprocessor component activity may be negatively regulated by HL, similar to the de-etiolation process. Our work provides deeper insight into the cross-talk between retrograde signaling and miRNA expression, creating new perspectives for further studies.

## Methods

### Plant materials

*Arabidopsis thaliana* Columbia-0 ecotype wild-type plants seeds were obtained from the Nottingham Arabidopsis Stock Centre (NASC, NASC code: N76778) and *ex1* (SALK_022735, NASC code: N522735) seeds. *flu* and *flu/ex1* seeds were kindly provided by C. Kim [67], while *mbs1* mutant seeds (SAIL_661_B05) were received from N. Shao [75]. The exact times at which plant materials were harvested for analysis are presented in Additional file 1: Figures S3, S6,S8, S10-S12.

## Hydroponic conditions

*A. thaliana* plants were grown for 4 weeks in hydroponic conditions [99] with some previously described modifications [13]. Briefly, seeds were surface sterilized using a chlorine gas method and kept for 2 days at 4 °C on agarose in high humidity to synchronize germination. Controlled growth conditions included a short-day photoperiod (8 h light/16 h dark), 22 °C/20 °C (day/night), 70% air humidity, and low light intensity (LL; 100–120 µmol m^−2^ s ^−1^).

## High-light treatment procedures

HL treatment was applied by exposing LL-adapted plants to high light (HL) intensity for 2 h using LED light sources (HL; 1500 µmol photons m^−2^ s^−1^; Photon Systems Instruments, Brno, Czech Republic).

## Chlorophyll a fluorescence

Chlorophyll a fluorescence parameters were determined using a PAM FluorCam 800 MF PSI device (Brno, Czech Republic). The plants were transferred to darkness for 30 min prior to measurement. The *F*_v_/*F*_m_ parameter, which reflects PSII maximum efficiency, was measured.

## Microtranscriptomic sequencing

Four-week-old *A. thaliana* plants grown in hydroponic conditions were subjected to HL stress. Plant material was sampled just after exposure to HL stress conditions (sample name: HL) and after recovery in LL conditions for 4 h (LLr). RNA isolation was performed using the Universal RNA/miRNA purification kit (EUR_X,_ cat. no E3599 Gdańsk, Poland) according to the manufacturer’s instructions. miRNA library preparation, miRNA sequencing and data analysis were performed by GENOMED S. A (Warsaw, Poland). Briefly, miRNA libraries were prepared using the NEBNext® Small RNA Library Prep Set for Illumina® (Multiplex Compatible) and sequenced using the Illumina HiSeq 4000 platform (Illumina Inc., San Diego, CA, USA). Bioinformatic analysis was also outsourced and conducted as previously described in Barczak-Brzyżek et al. 2019 [13]. Briefly, quality control checks of raw sequence data were performed using the FASTQ tool. Then, for trimming adapters, the Cutadapt program was used with subsequent identification of novel and known miRNAs using miRDeep2. Finally, the EdgeR Bioconductor package was applied for differential expression analysis. Microtranscriptomic screening results are presented for 3 independent experiments. In all cases, each biological replicate was pooled from six plants. The trimmed sequence data were deposited in the SRA database under accession number PRJNA650313.

## Chromatin immunoprecipitation (ChIP)

4-week-old *A. thaliana* Col-0 plants grown in hydroponic conditions were subjected to 2 h of HL treatment. Plant material was harvested to analysis at 13^30^. In all cases, each biological replicate was pooled from approx. 20 plants. Chromatin immunoprecipitation was performed as described in Godoy-Herz et al. 2019 [79]. IP buffer was prepared based on Kaufmann et al. 2010 [100]. Plant material was crosslinked using formaldehyde and then grinded with liquid nitrogen. Subsequently, chromatin was isolated and then sonicated before proceeding to immunoprecipitation. Next antibodies against total Pol II (Agrisera AS11 1804) were used with Dynabeads Protein G (Invitrogen, cat.no 10003D). Chelex (Biorad, cat.no 1421253) was used for de-crosslinking as described in Nelson et al. 2009 [101]. No antibody control was used to determine nonspecific background and percentage of input was calculated for each sample using qPCR.

## RNA stability assay

A cordycepin RNA stability assay was performed as described before in Fedak et al. 2016 [102]. Arabidopsis seedlings were grown for 2 weeks in LL (SD; 8 h light/16 h dark, temperature 22 °C/20 °C) on Murashige and Skoog (½ MS) medium (Duchefa Biochemie, cat. no M0222), supplemented with 1% w/v Sucrose (Duchefa Biochemie, cat. no S0809 and 0.7% phytoagar (Duchefa Biochemie, cat. no P1003), pH 5.7. Seeds were stratified at 4 °C for 2 days after sowing on Petri dishes. Seedlings were kept in LL (growth chamber) or HL conditions (1 h). Plant material was harvested to analysis from 12^10^ to 13^30^. Seedlings were collected and transferred to a flask containing incubation buffer (1 mM Pipes, pH 6.25, 1 mM trisodium citrate, 1 mM KCl, 15 mM sucrose. After 15 min of incubation, cordycepin was added to a final concentration of 150 µg/mL and vacuum-infilitrated (approx.2 × 5 min). At each time point (0, 20, 40, 60, 80 min), seedlings representing approx. 0.05 g were collected and frozen in liquid nitrogen. Samples were analyzed in triplicate. RNA extraction was performed using TRIzol method. RT-qPCR. analysis was performed with primers listed in Addifional file 1:Table S2. Calculation of pri-miRNAs half-life was performed as described in Chen et al. 2008 [103].

## DCMU and DBMIB treatments

Four-week-old *A. thaliana* plants grown in hydroponic conditions (see above) were treated with inhibitors. DCMU and DBMIB stock solutions (30 mM) were prepared by dissolving DCMU (3-(3,4-dichlorophenyl)-1,1-dimethylurea, Sigma Aldrich cat. no D2425) and DBMIB (2,5- dibromo-6-isopropyl-3-methyl-1,4-benzoquinone, Sigma Aldrich cat. no 271993) in DMSO (dimethyl sulfoxide, Sigma Aldrich cat. no 8418). DCMU and DBMIB working solutions were prepared by diluting appropriate stock solutions with sterilized water to a final concentration of 30 µM. For control treatments, plants were sprayed with 0.1% DMSO solution. In all cases, each biological replicate was pooled from six plants.

## EX1-dependent ^1^O_2_ signaling study

For the results presented in Fig. 3a-c *ex1*, *flu* and *flu/ex1* plants were grown for 2 weeks on 90 mm diameter Petri dishes on half-strength Murashige and Skoog (½ MS) medium (Duchefa Biochemie, cat. no M0222) supplemented with 1% w/v sucrose (Duchefa Biochemie, cat. no S0809 and 0.7% phytoagar (Duchefa Biochemie, cat. no P1003), pH 5.7. Induction of ^1^O_2_ accumulation was achieved in *flu* background plants by transferring plants cultivated in CL (constant light: LL intensity 90–110 µmol m^−2^ s ^−1^, temperature 20 °C humidity 70%) to the dark for 12 h followed by 2 h reillumination. In all cases, each biological replicate was pooled from at least six plants.

For the results presented in Fig. 3d, 4-week-old *A. thaliana* Col-0 and *ex1* plants grown in hydroponic conditions were subjected to HL treatment. In all cases, each biological replicate was pooled from six plants.

## β-CC-dependent ^1^O_2_ signaling study

For the results presented in Fig. 4b-d, 3- to 5-week-old *A. thaliana* plants were grown in pots under controlled conditions with a long photoperiod (light intensity approx. 250 µmol photons m^−2^ s^−1^, temperature 20 °C and humidity 70%). β-CC (β-cyclocitral; Santa Cruz Biotechnology, cat no sc-207467) treatment was performed as previously described [30]. Briefly, plants were placed for 4 h in a transparent plexiglass box (approx. 15 l vol.) with defined volumes (50 µl and 1 ml) of β-CC applied on a cotton wick to increase the contact area with the air. For the control conditions, the β-CC was replaced by distilled water. In all cases, each biological replicate was pooled from six plants.

For the results presented in Fig. 4e, 4-week-old *A. thaliana* Col-0 and *mbs1* plants grown in hydroponic conditions were subjected to HL treatment. In all cases, each biological replicate was pooled from six plants.

## RNA preparation

RNA extraction was performed using the Universal RNA/miRNA purification kit EUR_X_ (EUR_X,_ cat. no E3599, Gdańsk, Poland) according to the manufacturer’s instructions (for results presented in Fig. 1b-e and Fig. 2c) or using TRIzol reagent (Invitrogen, cat. no 15596062) (for results presented in Fig. 3b-d and Fig. 4b-e). The obtained RNA was treated with TurboDNase (Invitrogen, cat. no AM2238) according to the manufacturer’s recommendation. The RNA concentration was estimated using a NanoDrop 1000 (Thermo Fischer Scientific, Wilmington, MA, USA).

## RT-qPCR for mRNA quantification

### cDNA synthesis

A Quantitect Reverse Transcription kit (Qiagen, cat. no 205313, Hindel, Germany) was used for cDNA synthesis (for results presented in Fig. 1e and Fig. 2c) according to the manufacturer’s instructions. Oligo dT-primed cDNA was prepared using Superscript III reverse transcriptase (Invitrogen cat. no 18080093; for results presented in Fig. 3b-d and Fig. 4b,c,e).

## Quantitative RT PCR

qRT-PCR was performed in triplicate using the Bio-Rad CFX96 Touch ™ Real-Time PCR Detection System (Bio-Rad, Hercules, CA United States)/Roche Light Cycler® 384 System (Basel, Switzerland) with the primers listed in Additional file 1: Table S2. Real-time PCR cycling conditions were optimized depending on the primer used in the protocol, and relative expression was calculated with reference to the *UPL7* (AT3G53090) and *PP2A* (AT1G13320) genes. Product melting curves were generated following PCR to ensure amplification product purity.

Reverse transcription for miRNAs was performed with the qScript flex cDNA synthesis kit (Quantabio, cat. no 95047, Beverly, USA) according to [46] in a 10 µl total reaction volume. RNA was diluted in TE-LPA buffer (TE buffer with Linear Poly-Acrylamide (Ambion®, cat no AM9520) at a final working concentration of 20 µg/mL). The RT reaction mixture contained 10 ng of total RNA, 1 × RT buffer, 0.05 µM RT gene-specific primer (see primers list Additional file 1: Table S2), 1 µl GSP enhancer, and 0.5 µl RT enzyme. RT reactions were incubated in PCR tubes for 45 min at 25 °C and 5 min at 85 °C and then held at 4 °C.

## Two-tailed qRT PCR (TT qRT PCR)

TT qRT PCR was performed according to Androvic et al. 2017 [46], as previously described in Barczak-Brzyżek et al. 2019 [13]. The primers used in the study were designed in collaboration with BIOCEV, Institute of Biotechnology CAS, Czech Republic. Specifically, 1 × SYBR, 0.4 µM forward and reverse primer (see primers list Additional file 1:Table S2), and the cDNA product diluted 5 × were mixed in a 10 µL total reaction volume. Reactions were performed in triplicate and incubated in 96-well plates (CFX 96 Real Time Detection System (Bio-Rad)) at 95 °C for 30 s, followed by 45 cycles of 95 °C for 5 s and 60 °C for 15 s. Reaction specificity was assessed by melting curve analysis. Expression levels were calculated relative to those of snoRNA85 (NCBI Accession Number AJ505658) and snoRNA101 (NCBI Accession Number AJ505631).

## Statistical analysis

Statistical analysis was performed in R (version 3.6.0).

## Supplementary Information

**Additional file 1.**

## Data Availability

The trimmed data were deposited in the SRA database under accession number PRJNA650313. All the other data supporting the results of this article are included within the paper and its supplementary file as figures or tables.

## Abbreviations

^1^O_2_: Singlet oxygen
ChIP: Chromatin immunoprecipitation
DBMIB: 2,5- Dibromo-6-isopropyl-3-methyl-1,4-benzoquinone
DCMU: 3-(3,4-Dichlorophenyl)-1,1-dimethylurea
DCL1: Dicer-like 1
EX1: Executer 1
GM: Grana margins
HL: High light
LL: Low light
MBS1: Methylene Blue Sensitivity 1
miRNAs: Micro RNAs
PAP: 3’-Phosphoadenosine 5’ phosphate
PCR: Polymerase chain reaction
PET: Photosynthetic electron transport
PolII: RNA polymerase II
PQ: Plastoquinone
PQH2: Plastoquinol
pri-miR: Primary micro RNAs
PSI: Photosystem I
PSII: Photosystem II
qRT PCR: Quantitative reverse transcription PCR
RISC: RNA induced silencing complex
ROS: Reactive oxygen species
sno85: Small nucleolar85 RNA
sno101: Small nucleolar101 RNA
SORGs: Singlet oxygen responsive genes
TF: Transcription factor
TT qRT PCR: Two-tailed qRT PCR
β-CC: β- Cyclocitral
