# Supplementary material for: Plastid retrograde regulation of miRNA expression in response to light stress
Source: BMC Plant Biol. 2022 Mar 26;22:150. doi: 10.1186/s12870-022-03525-9 (PMC8962581; doi:10.1186/s12870-022-03525-9)
Supplement: Supplementary file 1 — Additional file 1. [file 12870_2022_3525_MOESM1_ESM.docx]

Additional file 1

Title: Plastid retrograde regulation of miRNA expression in response to light stress

Authors: Anna Barczak-Brzyżek^a^, Grzegorz Brzyżek^b^, Marek Koter^a^, Ewa Siedlecka^a^, Piotr Gawroński^a^, Marcin Filipecki^a,*^

Affiliations:

^a^ Department of Plants Genetics. Breeding and Biotechnology, Institute of Biology, Warsaw University of Life Sciences-SGGW, 02-776 Warsaw, Poland

^b^ Institute of Biochemistry and Biophysics Polish Academy of Sciences, 02-106 Warsaw, Poland

email: marcin_filipecki@sggw.edu.pl

* author to whom correspondence should be addressed

ORCID ID ABB: 0000-0003-2541-427X, GB: 0000-0002-6439-4462, MF: 0000-0003-4107-2484, PG: 0000-0002-9773-3109, MK: 0000-0002-9821-8475, ES: 0000-0002-7858-2843

**Table S1.** Selected miRNAs targeted transcripts of nuclear encoded proteins localized in chloroplasts.

| **miRNA** | **target accession** | **target name** | **target description** |
| --- | --- | --- | --- |
| ath-miR163 | at2g47940 | DEG2 | encodes chloroplastic DegP-type serine protease 2 |
| ath-miR395b/c/f | at3g22890 | APS1 | encodes ATP sulfurylase. the first enzyme in the sulfate assimilation pathway of Arabidopsis |
|  | at5g43780 | APS4 | encodes sulfate adenylyltransferase. ATP sulfurylase |
| ath-miR5021 | at3g60750 | TKL1 | encodes transketolase |
|  | at2g19450 | TAG1 | encodes Acyl-CoA:diacylglycerol acyltransferase (DGAT) catalyzes the final step of the triacylglycerol synthesis pathway |
| ath-miR414 | at3g52230 | hypothetical protein | function unknown |
| ath-miR5998a/b | at4g33010 | GLDP1 | encodes glycine decarboxylase P-protein 1 |

**Table S2. List of primers used in this study.**

| **Primer name** | **Accession** | **Primer sequence 5’-3’** | **Application** |
| --- | --- | --- | --- |
| PP2AA3_F | **AT1G13320** | TAACGTGGCCAAAATGATGC | qRT-PCR - reference |
| PP2AA3_R |  | GTTCTCCACAACCGCTTGGT | qRT-PCR - reference |
| UPL7_F | **AT3G53090** | TTCAAATACTTGCAGCCAACCTT | qRT-PCR - reference |
| UPL7_R |  | CCCAAAGAGAGGTATCACAAGAGACT | qRT-PCR - reference |
| APX2_F | **AT3G09640** | TCATCCTGGTAGACTGGACAAA | qRT-PCR |
| APX2_R |  | CACATCTCTTAGATGATCCACACC | qRT-PCR |
| CAT2_F | **AT4G35090** | TCTGGTGCTCCTGTATGGAA | qRT-PCR |
| CAT2_R |  | TGGTAATCCTCAAGAAGGATAGGA | qRT-PCR |
| RRTF1_F | **AT4G34410** | TCGGGTATGCATTATCCTAACA | qRT-PCR |
| RRTF_R |  | AAGCTCTTGCTCCGGTGA | qRT-PCR |
| ELIP1_F | **AT3G22840** | GCACAAAGTTTAGCGACTTGC | qRT-PCR |
| ELIP1_R |  | CGCAACGAATCCAACCAT | qRT-PCR |
| DRP_F | **AT1G57630** | CAAACAGGCGATCAAAGGAT | qRT-PCR |
| DRP_R |  | CAACACCACGAAGAAGCGTA | qRT-PCR |
| ath-sno85_RV1 | **AJ505658** | GTGCATTCAAAAGCCCTTACA | TT-qRT-PCR - reference |
| ath-sno85_FW1 |  | GCTTTGAAAGAGAGAGAGAGAG | TT- qRT-PCR - reference |
| ath-sno101_RV1 | **AJ505631** | GTTGATAACTACTGGTCTGCTGAT | TT- qRT-PCR - reference |
| ath-sno101_FW1 |  | TGTGAAGAGAGAGAGAGAGAG | TT- qRT-PCR - reference |
| ath-miR163_RV1 | **AT1G66725** | TTGAAGAGGACTTGGAACTTC | TT- qRT-PCR |
| ath-miR163_FW2 |  | GGTCCTCTAAGAACCACAGAG | TT- qRT-PCR |
| ath-miR840-5p_RV1 | **AT2G02741** | CGACACTGAAGGACCTAAACT | TT- qRT-PCR |
| ath-miR840-5p_FW2 |  | CCTTCAGCACACACACAGAC | TT- qRT-PCR |
| ath-miR395a/d/e_RV1 | **AT1G26973**  **AT1G69792**  **AT1G69795** | CTGAAGTGTTTGGGGGAA | TT- qRT-PCR |
| ath-miR395a/d/e_FW1 |  | GGCGACACTTCAATATACACAGG | TT- qRT-PCR |
| ath-miR5021_RV1 | **AT2G08235** | CGCTGAGAAGAAGAAGAAGA | TT- qRT-PCR |
| ath-miR5021_FW2 |  | TTCTTCTAGTGTCCCCGAC | TT- qRT-PCR |
| ath-miR165a-5p_RV1 | **AT1G01183** | GCGGAATGTTGTCTGGATCG | TT- qRT-PCR |
| ath-miR165a-5p_FW1 |  | ACATTCCCACACTCACAGAC | TT- qRT-PCR |
| ath-miR167b-5p_RV1 | **AT3G63375** | TGAAGCTGCCAGCATGAT | TT- qRT-PCR |
| ath-miR167b-5p_FW1 |  | AGCTTCACACACACACAGAC | TT- qRT-PCR |
| ath-miR319b_RV1 | **AT5G41663** | TTGGACTGAAGGGAGCTC | TT- qRT-PCR |
| ath-miR319b_FW1 |  | GTCAGTCAAGCAGAGAAGAG | TT- qRT-PCR |
| ath-miR390b-3p_RV1 | **AT5G58465** | GCCGCTATCCATCCTGAGT | TT- qRT-PCR |
| ath-miR390b-3p_FW1 |  | CGGATAGCGAAGAACAACAGAG | TT- qRT-PCR |
| RT-ath-snoR85_1 | **AJ505658** | TTTGAAAGAGAGAGAGAGAGCTAGAGAACCTAGCTCAATAGGAAGACATGT | RT reaction TT- reference |
| RT-ath-snoR101_1 | **AJ505631** | TGTGAAGAGAGAGAGAGAGAGCTAGAGAACCTAGCTCATTAGGAAGAGCATC | RT reaction TT- reference |
| RT-ath-miR163_2 | **AT1G66725** | GTCCTCTAAGAACCACAGAGCTAGAGAACCTAGCTCAACAACCACATCGAA | RT reaction TT |
| RT-ath-miR840-5p_2 | **AT2G02741** | CCTTCAGCACACACACAGACGTAGAGAACCTACGTCCACCATACCGTTAGT | RT reaction TT |
| RT ath-miR395a/d/e_1 | **AT1G26973** | ACACTTCAATATACACAGGAATTGAGGCATTCCTGATCCCTTAGAGTTC | RT reaction TT |
|  | **AT1G69792** |  | RT reaction TT |
|  | **AT1G69795** |  | RT reaction TT |
| RT ath-miR5021_2 | **AT2G08235** | TTCTTCTAGTGTCCCCGACGTAGGTTGACTACGTCATCATCCAATTTTCT | RT reaction TT |
| RT-ath-miR165a-5p_1 | **AT1G01183** | ACATTCCCACACTCACAGACGTAGAGAACCTACGTCAACAATACACCTCGA | RT reaction TT |
| RT-ath-miR167b_1 | **AT3G63375** | AGCTTCACACACACACAGACGTAGAGAACCTACGTCCACCACACATAGATC | RT reaction TT |
| RT-ath-miR319b_1 | **AT5G41663** | TCAGTCAAGCAGAGAAGAGCTAGAGAACCTAGCTCAAGACCAATAGGGAG | RT reaction TT |
| RT-ath-miR390b-3p_1 | **AT5G58465** | GATAGCGAAGAACAACAGAGCTAGAGAACCTAGCTCAACAACACAGGAACT | RT reaction TT |
| ath-sno85_R1 | **AJ505658** | GGTCCAGTTTTTTTTTTTTTTTACATGTA | qRT-PCR – reference (Fig.S4) |
| ath-sno85_F1 |  | GGTGCATTCAAAAGCCCTT | qRT-PCR – reference (Fig.S4) |
| ath-sno101_R1 | **AJ505631** | GACCAGTAGTTATCAACAAGCGA | qRT-PCR – reference (Fig.S4) |
| ath-sno101_F1 |  | ACACTTGATCTCTGAACTTCACA | qRT-PCR – reference (Fig.S4) |
| ath-miR163F_3 | **AT1G66725** | gcagttgaagaggacttggaa | qRT-PCR (Fig.S4) |
| ath-miR163R_3 |  | ggtccagtttttttttttttttatcgaa | qRT-PCR (Fig.S4) |
| ath-miR840-5pF_1 | **AT2G02741** | gcagacactgaaggacct | qRT-PCR (Fig.S4) |
| ath-miR840-5pR_3 |  | gtccagtttttttttttttttgttagtt | qRT-PCR (Fig.S4) |
| pri-miR163_F | **AT1G66725** | CGGttCCtGAGAGtGAGtCC | qRT-PCR |
| pri-miR163_R |  | TCGaCCGTGCTCTTCCTaaG | qRT-PCR |
| pri-miR840_F | **AT2G02741** | tGGAAGACACtGAAGGACCt | qRT-PCR |
| pri-miR840_R |  | GaTaaaGaGaTCaTCGTGCGGa | qRT-PCR |
| pri-miR319b_F | **AT5G41663** | TCTTCGGTCCACTCATGGAG | qRT-PCR |
| pri-miR319b_R |  | CTCCCTTCAGTCCAAGCATA | qRT-PCR |
| AT1G66725_1F | **AT1G66725** | caattttgttcgtgtgtggtg | ChIP-qPCR |
| AT1G66725_1R |  | tggggtagtgtgtcgttgtc | ChIP-qPCR |
| AT1G66725_2F |  | aggcgtccatggattatcac | ChIP-qPCR |
| AT1G66725_2R |  | tccaccaatcaagacctatgc | ChIP-qPCR |
| AT1G66725_3F |  | AACTTCCTCCAGGCAGATGA | ChIP-qPCR |
| AT1G66725_3R |  | TAAATCCCCAAATGGGTTCA | ChIP-qPCR |
| AT1G66725_4F |  | CCCGTGTtttgtccagtttc | ChIP-qPCR |
| AT1G66725_4R |  | tgtgcatgacttacgttatctcttt | ChIP-qPCR |
| AT2G02741_1F | **AT2G02741** | AATGGAGCTGGATTCTCTGG | ChIP-qPCR |
| AT2G02741_1R |  | CTTCCTTGCTCGGTTCATGT | ChIP-qPCR |
| AT2G02741_2F |  | TGTAATACCCCGCACACTGA | ChIP-qPCR |
| AT2G02741_2R |  | GACTCGGGTCTCGTAAAGCA | ChIP-qPCR |
| AT2G02741_3F |  | ggaaagaaaagcagcagcag | ChIP-qPCR |
| AT2G02741_3R |  | ttgcttttgaatgaatacagattg | ChIP-qPCR |
| UBC1 | **AT2G02760** | CTGCGACTCAGGGAATCTTCTAA | RNA stability assay |
| UBC2 |  | TTGTGCCATTGAATTGAACCC | RNA stability assay |
| At3g45970-1 | **AT3G45970** | GTATCCACCGGTTACTACGAACCTG | RNA stability assay |
| At3g45970-2 |  | CAAGTCGGTTCATCGCCAAATTGGG | RNA stability assay |

**
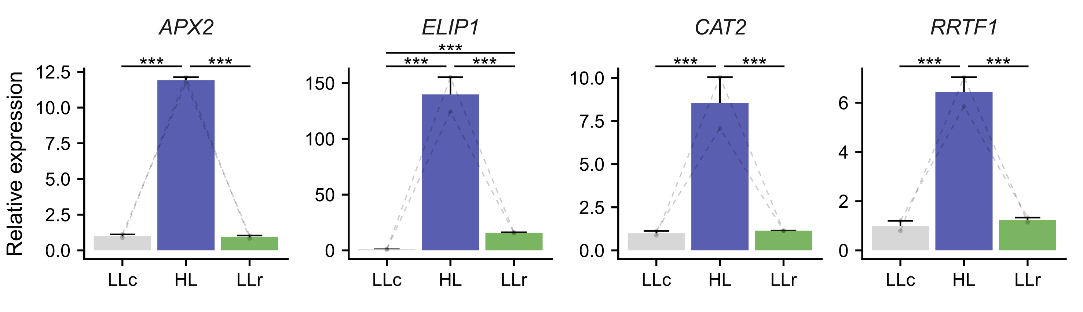
**

**Fig. S1** qRT-PCR for *APX2*, *ELIP1*, *CAT2* and *RRTF1*. Plant material: LLc - control plants; HL - plants exposed to HL for 2h; LLr - plants exposed to HL for 2 h, and a subsequent recovery for 4h in LL. Transcript levels were normalized with respect to the *PP2A* and *UPL7* genes. Asterisks indicate significant differences according to Tukey’s HSD test at the level of *** ≤ 0.001. Mean values ±SDs (n=2), were provided. In all cases, each biological replicate was pooled from six plants.


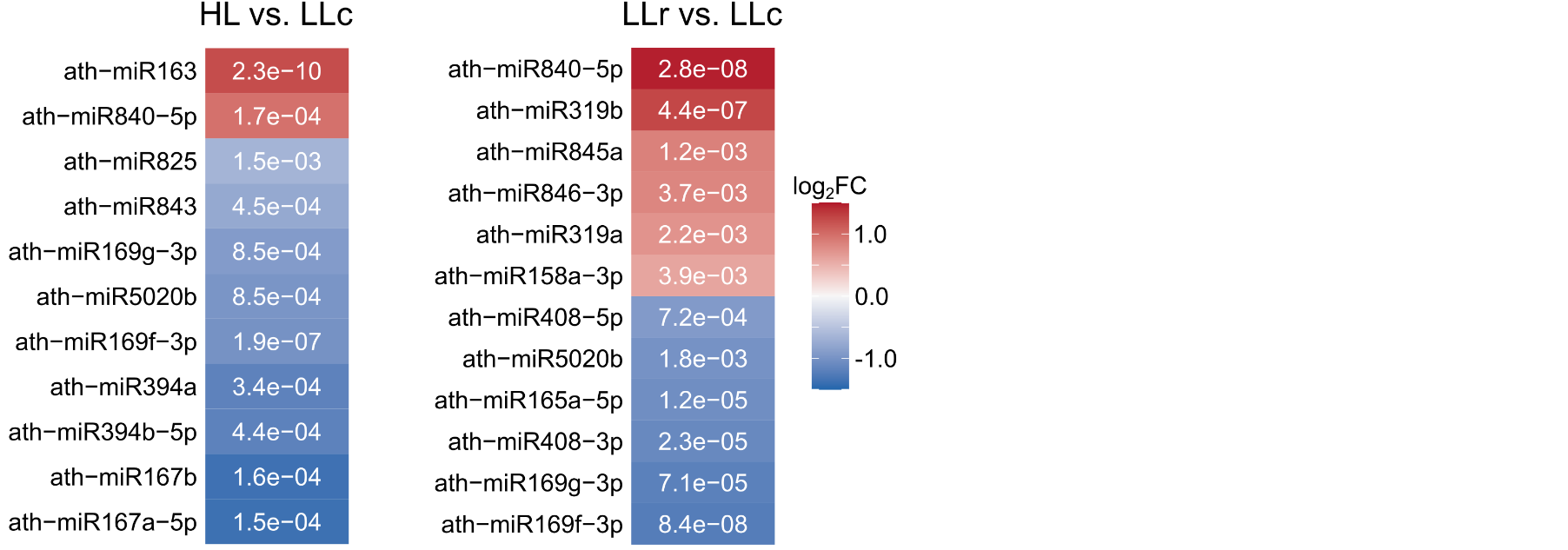


**Fig. S2** Heatmap represents miRSeq results for expression changes upon HL treatment. LLc - control plants; HL - plants exposed to HL for 2 h; LLr - plants exposed to HL for 2 h, and a subsequent recovery for 4h in LL.


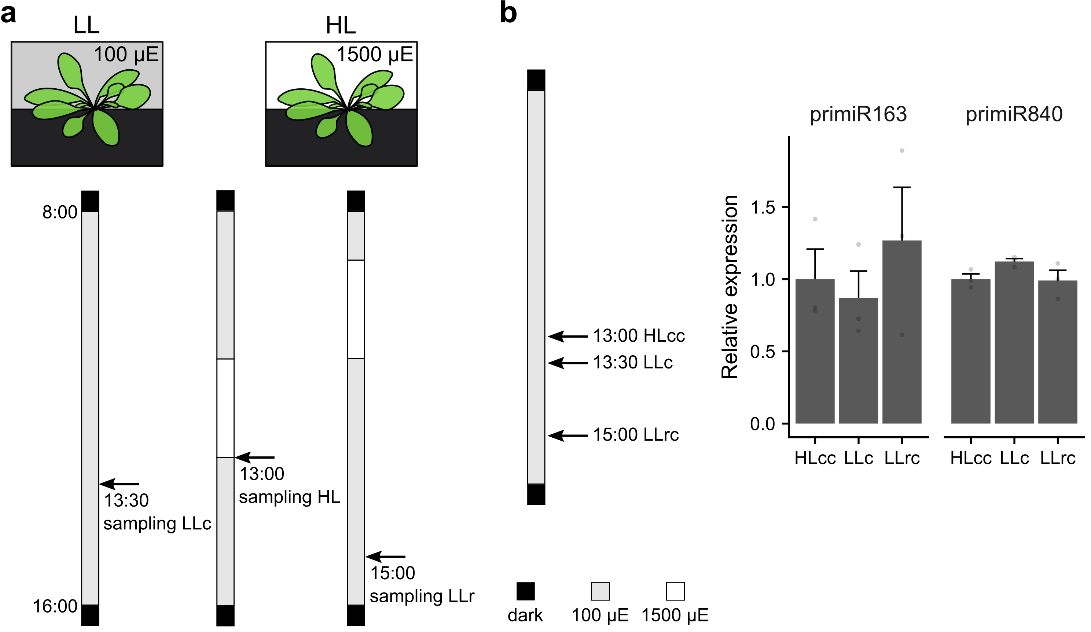


**Fig. S3 a** The scheme for the experiment represents times of sampling for miRSeq analysis; LLc - control plants; HL - plants exposed to HL for 2 h; LLr - plants exposed to HL for 2 h, and a subsequent recovery for 4h in LL. **b** The uniformity of expression of pri-miR163 and pri-miR840 in plants grown in control conditions collected in the time corresponding to LLc-LLcc, HL-HLc and LLr-LLrc, respectively. Transcripts level were normalized to the *PP2A* and *UPL7* genes. Significant differences were performed according to Tukey’s HSD test. Mean values ±SDs (n=3), were provided.


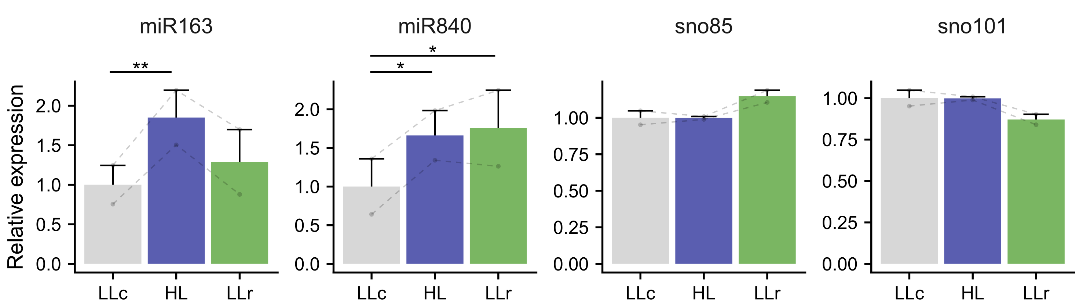


**Fig. S4** HL induces miRNA expression changes in *Arabidopsis thaliana* shoots. Validation of miRSeq data using qRT PCR based on Mir-X™ miRNA First Strand Synthesis kit (Takara, cat. no 638315; for details see (1)). LLc - control plants; HL - plants exposed to HL for 2 h; LLr - plants exposed to HL for 2 h, and a subsequent recovery for 4h in LL. Transcript levels were normalized with respect to sno85 and sno101. Asterisks indicate significant differences according to Tukey’s HSD test at the level of * ≤ 0.05. **≤ 0.01. Mean values ±SDs (n=2), were provided. In all cases, each biological replicate was pooled from six plants.


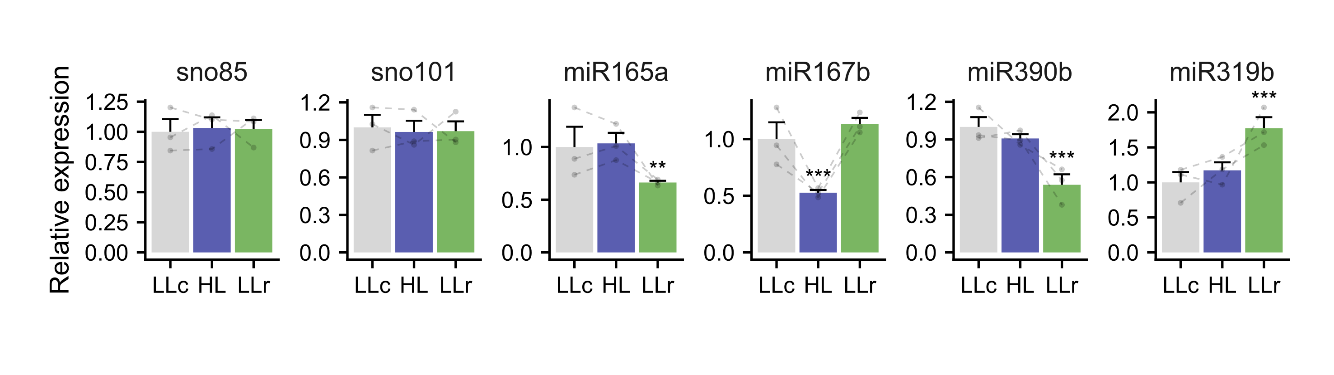


**Fig. S5** HL induces miRNA expression changes in *Arabidopsis thaliana* shoots. Validation of miRSeq data using a TT-qRT PCR. Transcript levels were normalized with respect to sno85 and sno101. Asterisks indicate significant differences according to Tukey’s HSD test at the level of **≤ 0.01, *** ≤ 0.001. Mean values ±SDs (n=3), were provided.


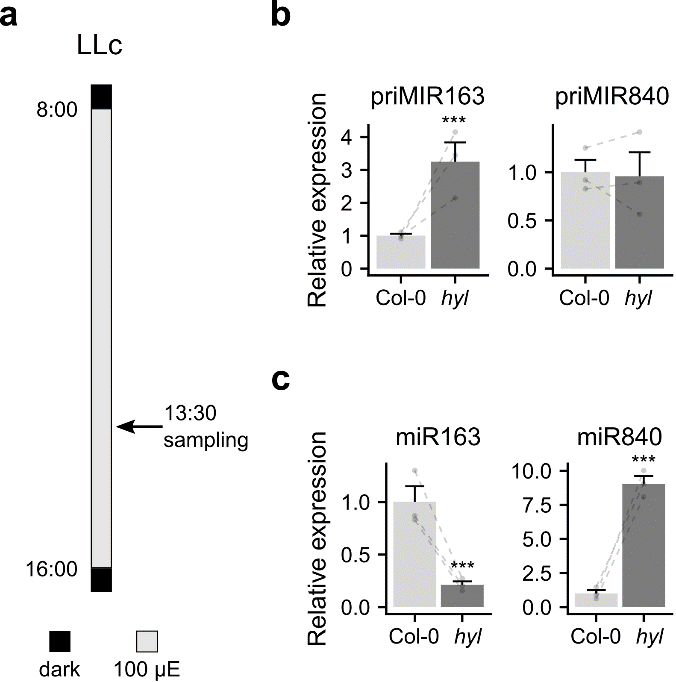


**Fig. S6 a** The experimental setup for plants used in the experiment with *hyl1* mutant. Seeds were obtained from NASC - *hyl1-2* (SALK_064863, NASC code: N859864) **b** qRT-PCR for pri-miR163 and pri-miR840 in Col-0 and *hyl1* mutant plants. Transcript levels were normalized with respect to the *PP2A* and *UPL7* genes. **c** TT-qRT-PCR for miR163 and miR840 in Col-0 and *hyl1* mutant plants. Transcript levels were normalized with respect to sno85 and sno101. Asterisks indicate significant differences according to the Tukey HSD test at the level of *** ≤ 0.001. Mean values ±SDs (n=3), were provided.


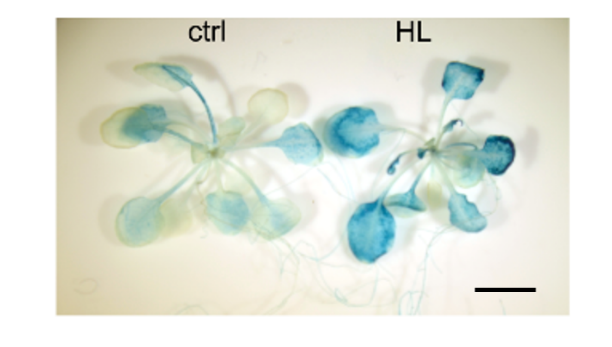


**Fig. S7** Representative GUS staining images of 4-week-old Arabidopsis plant exhibits pri-miR163 promoter fusion with beta-glucoronidase, grown in LL (left) or exposed to HL for 2h (right) . Scale bar represents 1 cm.


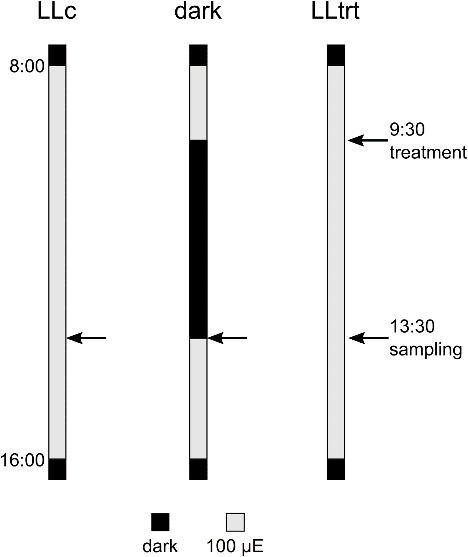


**Fig. S8** The experimental setup for plants used in the experiment with PET inhibitors (Figure 2). Plants were grown in short photoperiod (8h light/16h dark). The light intensity was set on 100-150 µE (LL) from 8^00^ to 16^00^. Plants were kept in the darkness for 4 h (dark) or treated with DCMU/ DBMIB and kept in LL for 4h (from 9^30^ to 13^30^; LLtrt); LLc – control plants kept in LL.


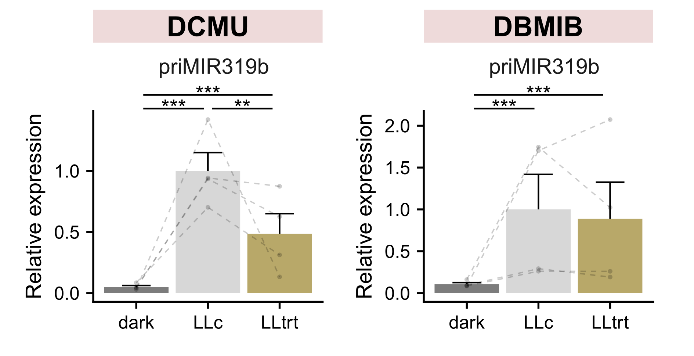


**Fig. S9** qRT-PCR for pri-miR319b after using DCMU or DBMIB. dark - plants kept in darkness for 4h; LLc - control plants in LL; LLtrt - plants treated with DCMU or DBMIB for 4h, kept in LL. Transcript levels were normalized with respect to the *PP2A* and *UPL7* genes. Asterisks indicate significant differences according to Tukey’s HSD test at the level of **≤ 0.01, *** ≤ 0.001. Mean values ±SDs (n=3), were provided.


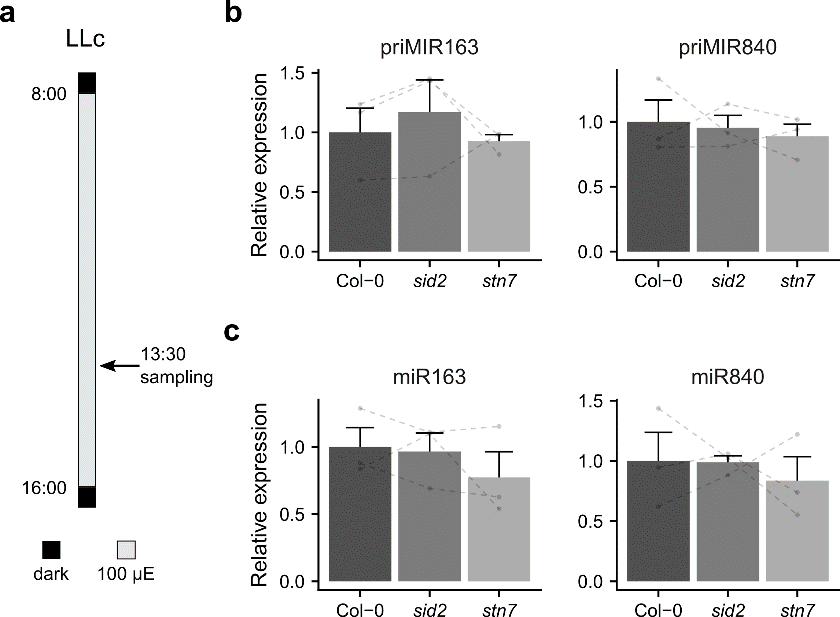


**Fig. S10** **a** The experimental setup for plants used in the experiment with PQ mutants **b** qRT-PCR for pri-miR163 and pri-miR840 in Col-0, *sid2-2* (deletion mutant derived from fast neutron bombardment mutagenesis – (2), NASC code N65996) and *stn7-1* ( SALK_073254, NASC code N573254) plants grown in LL. Transcript levels were normalized with respect to the *PP2A* and *UPL7* genes. **c** TT-qRT PCR for miR163 and miR840 in Col-0, *sid2-2* and *stn7-1* plants. Transcript levels were normalized with respect to sno85 and sno101. Significant differences were performed according to Tukey’s HSD test. Mean values ±SDs (n=3), were provided.


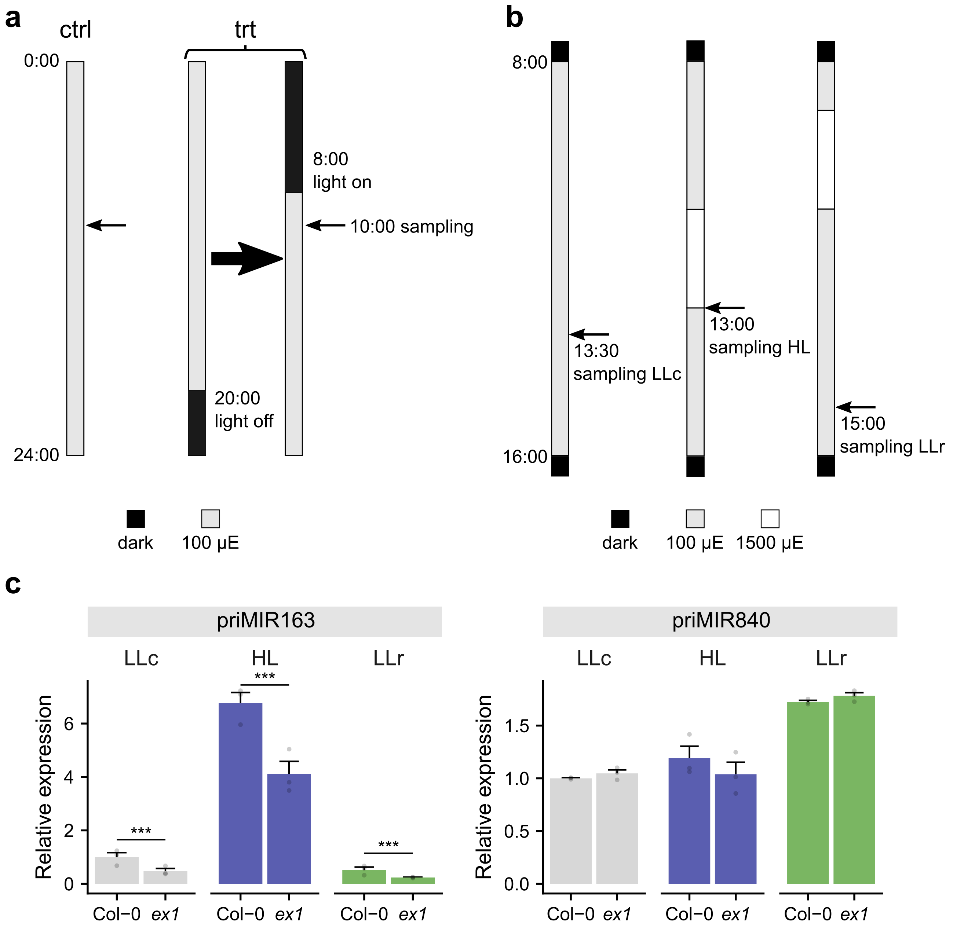


**Fig. S11** **a** The experimental setup represents time of sampling for plants used in Figure 3a-c; control plants - grown in constant light (ctrl), and treated plants (trt)- plants grown for 2 weeks in CL, placed for 12 h in darkness, and re-exposed for 2 h to LL **b** The experimental scheme for Figure 3d; LLc - control plants; HL - plants exposed to HL for 2 h; LLr - plants exposed to HL for 2 h, and a subsequent recovery for 4h in LL. **c** qRT-PCR for pri-miR163 and pri-miR840 in Col-0 and *ex1* plants; LLc - control plants; HL- plants exposed to HL for 2h; LLr - plants exposed to HL for 2h and subsequent recovery in LL for 4h. Transcript levels were normalized with respect to the *PP2A* and *UPL7* genes. Asterisks indicate significant differences according to Tukey’s HSD test at the level of *** ≤ 0.001.


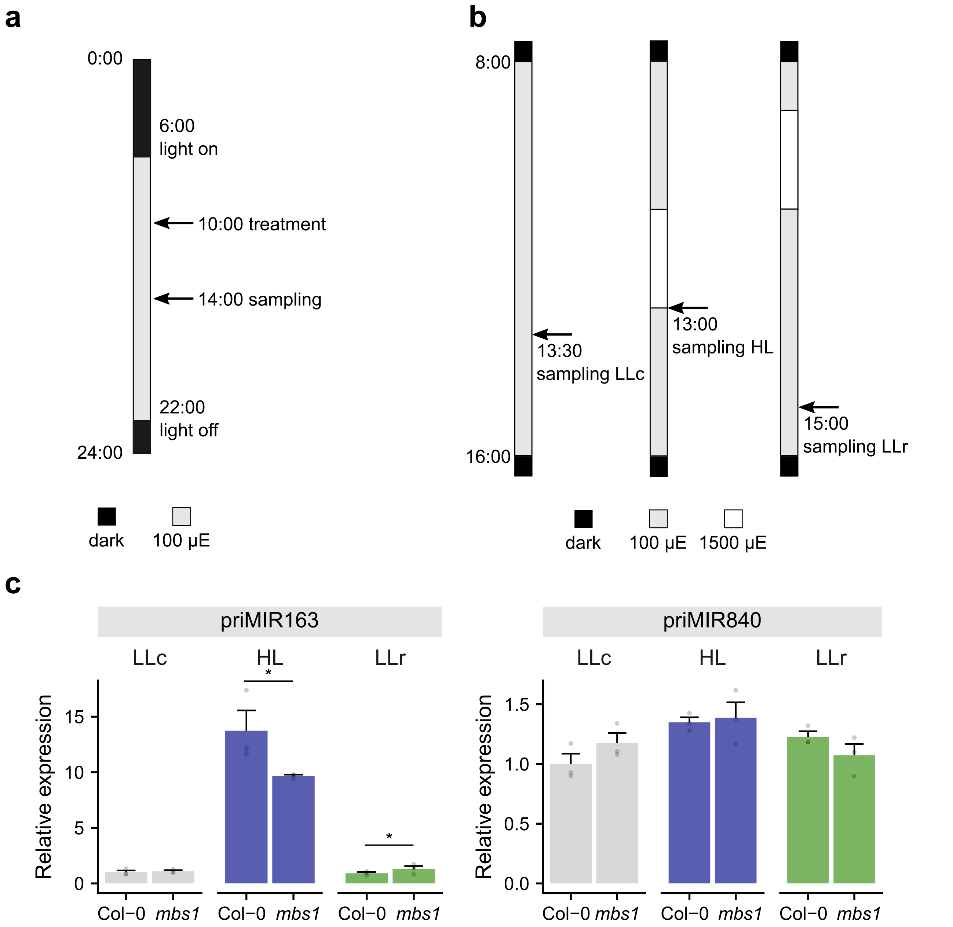


**Fig. S12** **a** The experimental setup represents time of sampling for plants used in Figure 4b-d **b** The experimental scheme for Figure 4e **c** qRT-PCR for pri-miR163 and pri-miR840 in Col-0 and *mbs1* plants; LLc-control plants; HL-plants exposed to HL for 2h; LLr-plants exposed to HL for 2h and subsequent recovery in LL for 4h. Transcript levels were normalized with respect to the *PP2A* and *UPL7* genes. Asterisks indicate significant differences according to Tukey’s HSD test at the level of * ≤ 0.05. Mean values ±SDs (n=3), were provided.

A


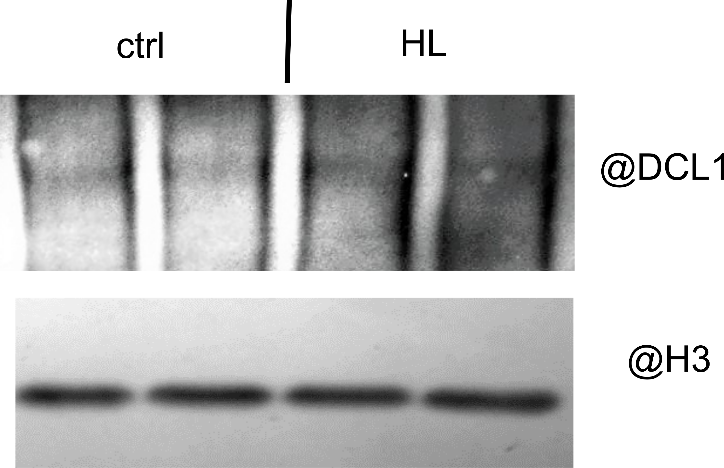


B


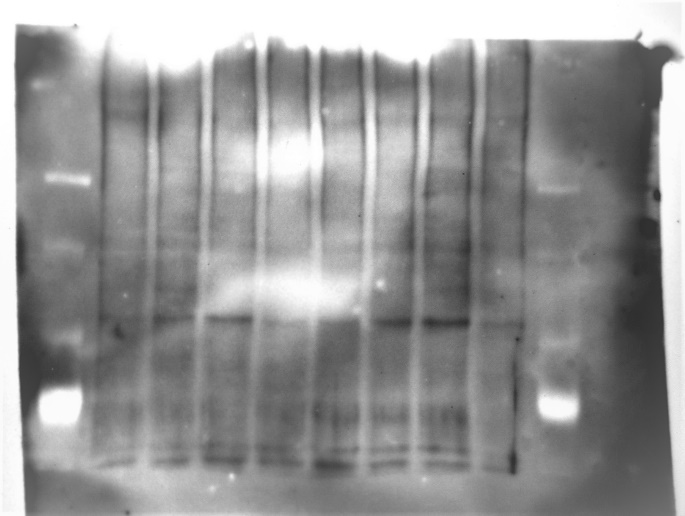


C


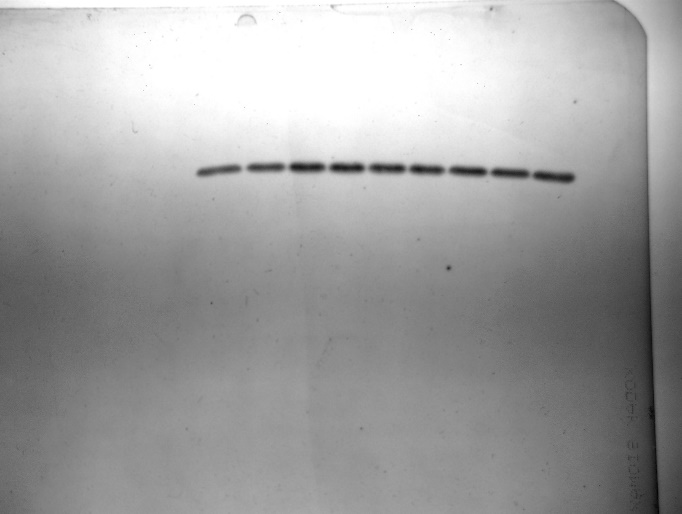


**Fig. S13** Detection of DCL1 in LL (ctrl) and HL conditions (HL) by Western blot analysis, histone H3 was used as a loading control ( for details see Supplementary methods). A – figure described in text. B and C – original blots.


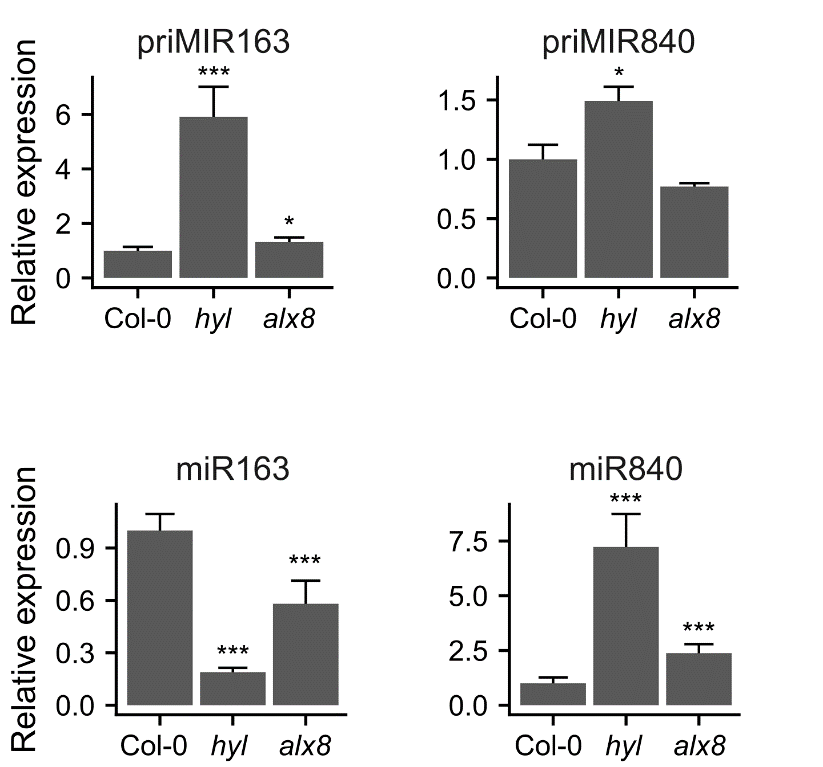


**Fig. S14** qRT-PCR for pri-miR163 and pri-miR840 in Col-0, *hyl1* and *alx8* (NASC ID:N66977; donated by The National Australian University by Barry Pogson) plants (upper panel). Transcript levels were normalized with respect to the *PP2A* and *UPL7* genes. Asterisks indicate significant differences according to the Tukey HSD test at the level of *≤ 0.05, *** ≤ 0.001. Mean values ±SDs (n=3), were provided. TT-qRT PCR for miR163 and miR840 in Col-0, *hyl1* and *alx8* plants (bottom panel). Transcript levels were normalized with respect to sno85 and sno101. Asterisks indicate significant differences according to Tukey’s HSD test at the level of*** ≤ 0.001. Mean values ±SDs (n=3), were provided. Plant material was harvested for analysis as presented in Fig. S9a.


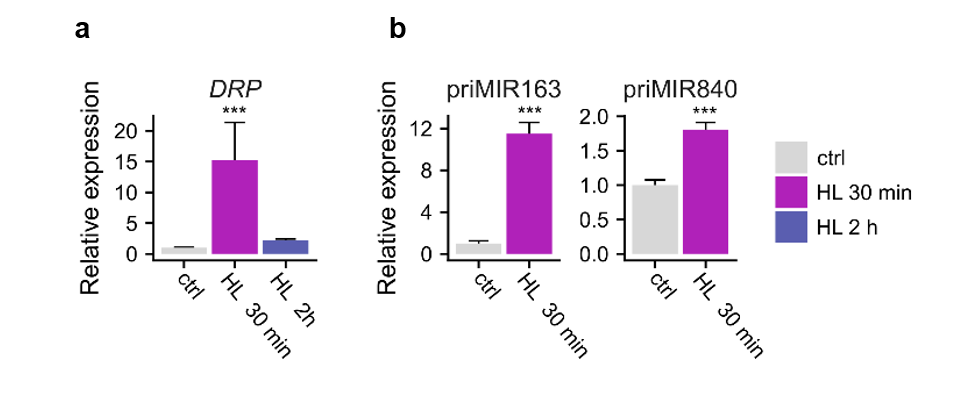


**Fig. S15 a** qRT-PCR for *DRP*, pri-miR163 and pri-miR840 – (**b)** in Col-0 LLc and HL30 min plants (plants exposed to HL for 30 min), HL 2h – plants exposed to HL for 2h . Transcript levels were normalized with respect to the *PP2A* and *UPL7* genes. Asterisks indicate significant differences according to Tukey’s HSD test at the level of *** ≤ 0.001. Mean values ±SDs (n=3), were provided.

**Supplementary methods**

**GUS histochemical staining**

4-week-old Arabidopsis plants (SD; 8h light/16h dark, temperature 22 °C/20 °C; grown in hydroponic conditions) were infiltrated with 50 mM sodium phosphate (pH 7.0), 10 mM EDTA, and 0.5mg/ml X-gluc (Duchefa Biochemie) and incubated at 37°C in the dark overnight. The plants were then rinsed in 70% ethanol until chlorophyll is removed. Pictures of representative plants were taken under a stereo microscope (Leica M165-FC; Leica Microsystems, Wetzlar, Germany).

**Analysis of DCL1 protein level**

**Nuclear proteins isolation**

4-week-old Arabidopsis plants (SD; 8h light/16h dark, temperature 22 °C/20 °C; grown on Jiffy pots) were frozen in liquid nitrogen (ctrl – plants grown in LL; HL- plants exposed to HL for 2h). Plant material was harvested to analysis at 13^30^. Collected plant tissue samples (0.5 g) were ground to a fine powder using mortar and pestle. Tissue powder was homogenised in 25 ml of ice cold nuclei isolation buffer (10 mM HEPES pH 7.5 , 1 M sucrose, 5 mM MgCl2, 5 mM KCl, 0.6% Triton X-100, 0.1% 2-mercaptoethanol, 0.4 mM PMSF) and incubated at 4 ^o^C for 15 min with gentle agitation to ensure the proper cell lysis. After the homogenate was filtered using one layer of Miracloth (Merck Milipore) to remove cell debris, filtered solution was spun down for 20 min, 3200 x g at 4 ^o^C and supernatant was removed. Pelleted nuclei were gently dissolved in 500 µl of fresh nuclei isolation buffer and pipetted on top of 800 µl nuclei separation buffer (10 mM HEPES pH 7.5 , 1 M sucrose, 5 mM MgCl2, 5 mM KCl, 5 mM EDTA, 15% Percoll, 1 mM PMSF, 1x EDTA free protease inhibitor cocktail (Roche)) in 1.5 ml eppendorf tube. Nuclei were centrifuged in percoll gradient at 4 ^o^C, 4000 x g for 5 min and the supernatant was carefully removed. Obtained nuclei were lysed using 100 µl lysis buffer (4 M urea, 1 mM MgCl_2_, 0.01% Triton X-100) and nucleic acids were digested 30 minutes at 4 ^o^C using 250 U Viscolase (A&A Biotechnology). Next nuclei debris were separated by by 5 min centrifugation at 20 000 x g. Obtained proteins were quantified using Bradford reagent, and equal amounts of proteins were mixed with 4x Leammli buffer.

**Western blotting**

Nuclear proteins were separated in SDS-PAGE gels and transferred to PVDF membrane. After blocking with 5% skimmed milk the membranes were immunoblotted with anti-histone H3 (Abcam ab1791) as a loading control and anti-DCL1 (Agrisera AS19 4307). Goat anti-Rabbit HRP conjugated antibodies (Agrisera AS09 602) were used subsequently. The chemiluminescent WesternBright™ Quantum system (Advansta) was used to develop the protein blots, signals were captured using photographic film.

**Supplementary references:**

1. Barczak-Brzyżek A., Brzyżek G, Koter M, Gawroński P, Filipecki M. Exposure to High-Intensity Light Systemically Induces Micro-Transcriptomic Changes in Arabidopsis thaliana Roots. Int J Mol Sci. 2019 Jan;20(20):5131.

2. Wildermuth MC, Dewdney J, Wu G, Ausubel FM. Isochorismate synthase is required to synthesize salicylic acid for plant defence. Nature. 2001 Nov;414(6863):562–5.
